# Strong Purifying Selection in Transmission of Mammalian Mitochondrial DNA

**DOI:** 10.1371/journal.pbio.0060010

**Published:** 2008-01-29

**Authors:** James Bruce Stewart, Christoph Freyer, Joanna L Elson, Anna Wredenberg, Zekiye Cansu, Aleksandra Trifunovic, Nils-Göran Larsson

**Affiliations:** 1 Department of Laboratory Medicine, Karolinska Institutet, Stockholm, Sweden; 2 Mitochondrial Research Group, The Medical School, University of Newcastle upon Tyne, United Kingdom; University of Bath, United Kingdom

## Abstract

There is an intense debate concerning whether selection or demographics has been most important in shaping the sequence variation observed in modern human mitochondrial DNA (mtDNA). Purifying selection is thought to be important in shaping mtDNA sequence evolution, but the strength of this selection has been debated, mainly due to the threshold effect of pathogenic mtDNA mutations and an observed excess of new mtDNA mutations in human population data. We experimentally addressed this issue by studying the maternal transmission of random mtDNA mutations in mtDNA mutator mice expressing a proofreading-deficient mitochondrial DNA polymerase. We report a rapid and strong elimination of nonsynonymous changes in protein-coding genes; the hallmark of purifying selection. There are striking similarities between the mutational patterns in our experimental mouse system and human mtDNA polymorphisms. These data show strong purifying selection against mutations within mtDNA protein-coding genes. To our knowledge, our study presents the first direct experimental observations of the fate of random mtDNA mutations in the mammalian germ line and demonstrates the importance of purifying selection in shaping mitochondrial sequence diversity.

## Author Summary

Mammalian mitochondrial DNA (mtDNA) is maternally transmitted and does not undergo bi-parental recombination in the germ line. This asexual mode of transmission, together with a high rate of mutation, should eventually lead to the accumulation of numerous deleterious mtDNA mutations and a “mutational meltdown” (a phenomenon know as Muller's Ratchet). In this study, we utilized a genetic mouse model, the mtDNA mutator mouse, to introduce random mtDNA mutations, and followed transmission of these mutations. Maternal transmission of mtDNA is typically subjected to a bottleneck phenomenon whereby only a fraction of the mtDNA copies in the germ-cell precursor are amplified to generate the approximately 10^5^ mtDNA copies present in the mature oocyte. As a consequence of this phenomenon, the established maternal mouse lines carried high levels of a few mtDNA mutations. We sequenced the entire mtDNA to characterize the maternally transmitted mutations in the established mouse lines. Surprisingly, mutations causing amino acid changes were strongly underrepresented in comparison with “silent” changes in the protein-coding genes. These results show that mtDNA is subject to strong purifying selection in the maternal germ line. Such selection of functional mtDNA genomes likely involves a mechanism for functional testing to prevent transmission of mutated genomes to the offspring.

## Abstract 3

We have used a genetic mouse model with a proofreading-deficient mitochondrial polymerase to mutagenize the mouse mitochondrial genome. The inherited mutations are subject to rapid purifying selection against amino acid substitutions.

## Introduction

Mammalian mitochondrial DNA (mtDNA) has a high mutation rate and is inherited in a non-Mendelian manner only from the mother [1,2]. Though there are reports of mitochondrial recombination in mammals, it is thought to be quite rare, and it is currently not known whether this phenomenon would be at a sufficient frequency to leave a signature in the population [3–6]. This asexual mode of transmission should leave the mitochondrial genome vulnerable to mutational meltdown by Muller's Ratchet, a process leading to deleterious mutation accumulation in asexual, nonrecombining lineages. The bottleneck phenomenon, which was first proposed after observation of rapid fixation of mitochondrial DNA variants in Holstein cows [7,8], allows for rapid exposure of variant mtDNAs to selection at the level of the individual [9], and may thereby, at the level of the population, protect against mutational meltdown.

The over 100,000 mtDNA molecules in the mammalian oocyte do not undergo replication through the early stages of embryogenesis [2]. Therefore, these maternally derived mtDNA molecules are segregated through cell division events in the developing embryo to generate primordial germ cells with approximately 950–1,550 mtDNA copies [10]. Replication of mtDNA is reinitiated as the primordial germ cells migrate and differentiate to generate oocytes transmitting mtDNA to the next generation [2,11]. The mtDNA bottleneck appears to result from the replication of only a small subset of the mtDNA molecules as the primordial germ cells differentiate to generate oocytes [10].

It has long been thought that animal mtDNA is an essentially neutral marker of sequence evolution [1], but evidence of the selective constraints on mtDNA is accumulating. Studies of animal mtDNA sequence variation within natural populations or in interspecies comparisons consistently show the signatures of negative selection (e.g., [12–14]). For humans, a considerable amount of mtDNA sequence is available from individuals as a result of studies into human evolution and human mtDNA diseases [15,16]. Analyses of the variation in human mtDNA sequences have led to a debate whether random genetic drift (dependent on demographic history), positive selection, or purifying selection is important in the transmission and maintenance of this variation. [17,18]. Consensus is forming that selection is an important part of mtDNA sequence variation in human mtDNA, but the strength and nature of this selection are unresolved [18]. Population-level studies detect signatures of purifying selection in mtDNA sequence variation [19–26], but recent accumulated variation within human populations implies neutrality or weak selection on these variants [25]. Findings from studies of mtDNA mutation inheritance in families with mtDNA-associated disease are compatible with the occurrence of only very weak or no selection on these mtDNA mutations [17,27]. Positive selection facilitated by climatic variation has recently been proposed for human mtDNA [28–30] and would profoundly affect the reliability of mitochondrial molecular clocks and drastically alter our understanding of human divergences.

In an attempt to elucidate the mechanisms of mammalian mtDNA segregation in the germ line, several groups generated transmitochondrial mouse strains carrying two distinct mtDNA sequences (a condition known as heteroplasmy). These transmitochondrial mice are generated by embryo–cytoplast fusions and exhibit germ line segregation patterns explainable by random drift [31–34]. However, tissue-specific segregation patterns within the offspring imply strong nuclear–mitochondrial interactions and suggest that molecular mechanisms exist that could allow for strong selection of mitochondrial variants within offspring [33,35–37]. Unfortunately, the technical complexities of the transmitochondrial technologies have much limited their use by research groups, and so far only a few sequence variants have been investigated.

The mtDNA mutator mice are homozygous for a knock-in allele (*PolgA^mut^*/*PolgA^mut^*) expressing a proofreading-deficient catalytic subunit of mitochondrial DNA polymerase [38]. These mice have a substantial increase in the levels of mtDNA mutation in all investigated tissues. The somatic mutations generated are evenly distributed along an amplified fragment of the protein-coding *mt-CYB* gene of mtDNA, and all three codon positions are mutated at equal frequency, though transition mutations were more frequently observed than transversions [38]. In this study, we took advantage of this high mtDNA mutation rate to study the transmission of random mtDNA mutations in the mouse germ line. Female lineages were derived from mtDNA mutator mice by continuous backcrossing, allowing us to isolate, segregate, and characterize germ line mtDNA mutations.

## Results

**Figure 1:**
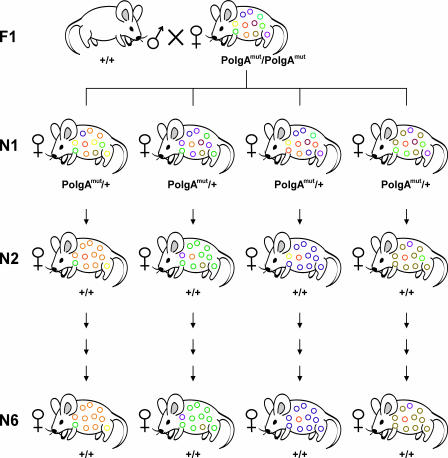
The Breeding Scheme for the Mutagenesis of Mouse mtDNA The *PolgA^mut^/PolgA^mut^* mtDNA mutator females were derived from mothers with wild-type C57Bl/6 mtDNA. The mtDNA mutators, and all subsequent generations, were crossed to wild-type C57Bl/6 males. Heterozygous (+/*PolgA^mut^*) N1 female progeny were used to establish the 13 mtDNA mutator lines. Only females that were homozygous for the wild-type *PolgA* allele (+/+) were bred from generation N2 and onwards.

We used eight mtDNA mutator founder females to establish independent maternal lines through 13 F1 females. The breeding scheme used (Figure 1) takes advantage of the bottleneck phenomenon and allowed us to segregate the mtDNA mutations on a wild-type *PolgA* nuclear background from generation N2 and onwards. Sequencing was conducted from N2 onwards to sample only animals of wild-type *PolgA* nuclear background, and because levels of individual mutations in mtDNA mutator mice and N1 animals were too low to be detected by the sequencing methods employed.

We sequenced the entire mtDNA of 190 animals from generations N2 to N6 and identified 1,069 unique mutations (Dataset S1). The typical animal carried approximately 30 mtDNA mutations (mean = 29.8 mutations, standard deviation = 9.2). In each line, a large proportion of the identified mtDNA mutations (38.48%) were transmitted to the descendents of that particular N1 female, similar to the propagation of mtDNA haplogroups in human pedigrees. Other mutations were only observed in the siblings of a single litter (17.98%) or in a single mouse (43.54%), but not in their offspring.

Consistent with purifying selection acting on the mtDNA of these mouse lines, synonymous mutations were observed more frequently than nonsynonymous mutations. The ratio of nonsynonymous substitutions per site to synonymous substitutions per site for the protein-coding regions gave a value of 0.6035, signifying purifying selection against amino acid changes in the protein-coding genes (values less than 1.0 signify purifying selection). When mutations that occur only in an individual or a single litter were removed from the dataset, the ratio dropped to 0.4617. The McDonald-Kreitman test of neutral evolution [39] and the accompanying Neutrality Index [40] were calculated for our mtDNA mutator lines, and gave values consistent with excess polymorphisms within our mtDNA mutator lines compared to either *Mus musculus molossinus* or the NZB mouse strain mtDNA sequences (see Table S1).

**Figure 2:**
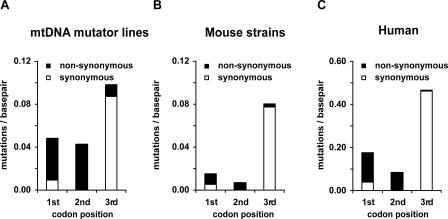
Mutation Distribution by Codon Position Reveals Purifying Selection on Protein-Coding Genes in mtDNA Mutator Lines Plot of observed mtDNA mutations grouped by positional category. (A) Observed mutations per base pair for each codon position from mtDNA mutator lines compared to (B), mtDNA sequences of 21 mouse strains obtained from GenBank, and (C), human mtDNA sequences obtained from the mtDB database. The reduction in observed first and second codon position mutations signifies selection against amino acid–changing mutations. Due to the larger number of human sequences available, the *y*-axis is larger for the human dataset.

We found a strong decrease in the number of mutations at the first and second codon positions of the protein-coding genes when compared to the third codon positions (Figure 2A and Table S2A). This distribution of mutations is a hallmark of purifying selection, because changes in the first and second codon position usually result in an amino acid substitution, whereas many third codon position changes do not. This purifying selection is strong and rapid as the same codon distribution bias is evident in the N2 generation (Figure S1). The observed nucleotide mutational bias in protein-coding genes varied significantly from those observed for the other sites in the mtDNA molecule (chi-square contingency table, *p* = 0.0023) thus showing differential selection pressures on the protein-coding genes versus other sites (Table 1).

**Table 1:**
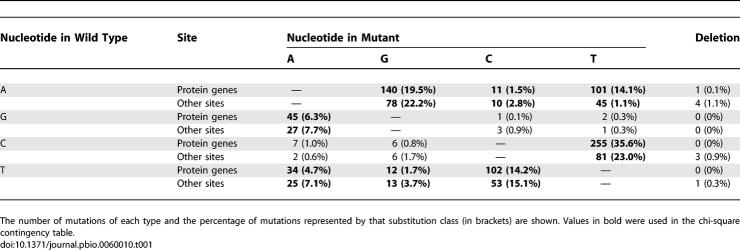
Observed Nucleotide Mutational Bias in Protein Coding and Non-Protein Coding Sites in mtDNA Mutator Lines

We observed the same selective signature against first and second codon positions when we compared 21 mouse-strain mtDNA sequences obtained from GenBank (Figure 2B) and human mtDNA sequence data obtained from the mtDB database [16] (Figure 2C and Table S2B). There was a similar level of reduction of first codon position mutations in comparison with third codon position mutations in mtDNA mutator lines (2.0-fold reduction; Figure 2A) and humans (2.6-fold reduction; Figure 2C). This striking similarity is surprising because the mtDNA mutator strains have undergone selection for at most six generations, whereas human sequence variation is the consequence of a much larger number of generations to act on these less deleterious substitutions. This illustrates the speed and strength of the selection on the mtDNA and its importance in sculpting modern mtDNA variation in natural populations. It also demonstrates this experimental model can be a powerful tool in investigation of mtDNA evolution. The smaller number of nonsynonymous changes in the mouse strains (Figure 2B) in comparison with mtDNA mutator lines (Figure 2A) can probably be explained by the limited sampling of only one individual from each of the 21 different mouse strains. In addition, it should be emphasized that the mtDNA mutator mice have been exposed to the effects of purifying selection for only a few generations.

**Figure 3:**
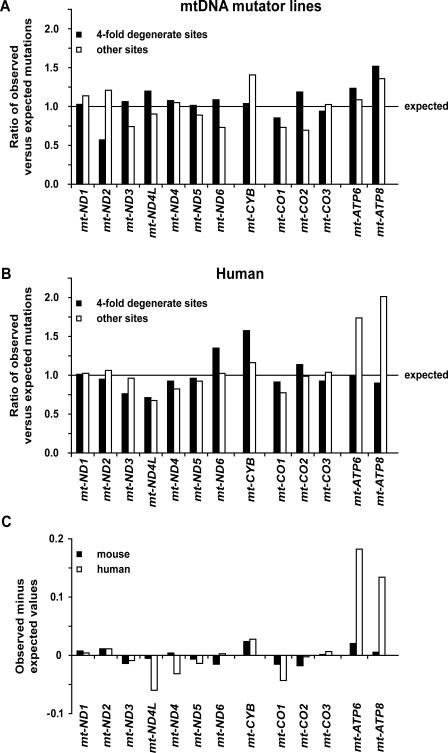
Distribution of Mutations by Gene for mtDNA Mutator Lines and Human mtDNA Sequence Data Plot of observed minus expected ratio of 4-fold degenerate sites versus all other protein-coding sites. (A) For mtDNA mutator mouse lines, the ratio of observed to expected sites is plotted for third codon position mutations at 4-fold degenerate sites (filled bars), and for all other protein-coding gene mutations observed (open bars). The line represents the observed expected values normalized to 1.0. (B) The same plot for observed human variants found on the mtDB database. (C) Plot of observed minus expected for non–4-fold degenerate sites for mouse (filled bar) and human (open bars). Genes are grouped by mitochondrial respiratory chain complexes. Expected values are derived from an assumption of equal distribution of the observed mutations in the dataset, and observed values are derived from a count of the detected mutations for that gene (see Material and Methods).

We further investigated the observed selection on protein-coding genes in the mtDNA mutator lines by separating the mutations at 4-fold degenerate sites (third codon positions for amino acids L2, V, A, T, P, S1, R, and G) from all other protein-coding mutations. The 4-fold degenerate sites can mutate to any nucleotide without changing the encoded amino acid and should therefore be subject to less selective constraint than other protein-coding sites. Expected values were calculated based on an assumption of an equal distribution of the observed mutations of these two classes, across the genes and corrected for their coding size. The ratios between observed and expected mutation frequencies at 4-fold degenerate sites were approximately equal in all of the protein-coding genes except for *mt-ND2* and *mt-ATP8* (Figure 3A and Table S3A). In contrast, mutations at the non–4-fold degenerate sites deviated profoundly from the ratios predicted by equal distribution of mutations (Figure 3A). Contingency table analysis was carried out to detect changes in the proportion of 4-fold degenerate site mutations to other sites within the protein-coding genes. The 13 protein-coding subunits were grouped by the oxidative phosphorylation (OXPHOS) enzyme complex to which they belong. Only complexes III (containing *mt-CYTB*) and complex IV (containing *mt-CO1–3*) showed statistically significant changes in the ratio of 4-fold to non–4-fold sites (see Table 2). After correcting for multiple tests, only the complex IV data remained significant. Interestingly, the *mt-ATP8* and *mt-ATP6* subunits appear to allow for excess changes at all sites relative to the expected values, though the ratio of 4-fold to non–4-fold sites did not vary significantly (Figure 3A). Analyses of human mtDNA sequences have shown a similar occurrence of excess sequence variation in the *mt-ATP8* and *mt-ATP6* genes, particularly evident for the *mt-ATP6* gene [23–25,28]. These previous reports lead us to investigate available human sequence data, and we found a strong selection against non–4-fold degenerate changes in *mt-CO1* versus the weaker selection in *mt-ATP6*, *mt-ATP8*, and *mt-CYTB* (Figure 3B and Table S3B). Observed mutations for each protein gene for mtDNA mutator lines and human population showed the same variation from expected in 11 of 13 cases (Figure 3C). Thus, sequence variation in protein-coding genes of this experimental mouse model demonstrates similar patterns to those seen in human populations.

**Table 2:**
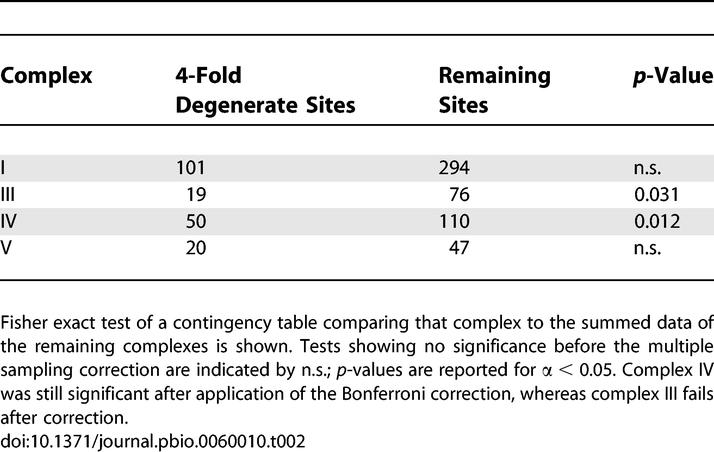
Comparison of 4-Fold Degenerate Sites to Other Sites for Each Mitochondrial-Encoded OXPHOS Enzyme Complex

**Figure 4:**
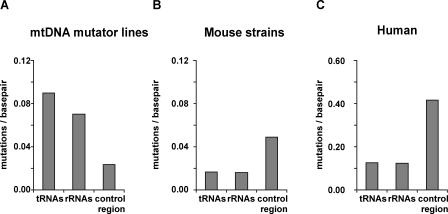
Relaxed Selection on rRNA and tRNA Genes in mtDNA Mutator Lines Plot of observed mtDNA mutations grouped by positional category. (A) Observed mutations in tRNA, rRNA, and control region sequences compared with (B) mouse strain sequences and (C) human mtDNA sequences. Due to the larger number of human sequences available, the *y*-axis is larger for the human dataset.

In contrast to the patterns found in the protein-coding genes, we found higher levels of mutations in tRNA and rRNA genes in the mtDNA mutator lines (Figure 4A) in comparison to the levels in mouse strains and humans (Figure 4B and 4C). There are several observations from human mtDNA disease that imply that tRNA genes may be subject to a less rapid form of purifying selection than that observed for the protein-coding genes of our mtDNA mutator mouse lines: (1) Population-level sampling has revealed an increase in recent mtDNA sequence variation within tRNA and rRNA genes in humans [30,41]; (2) 58.2% of the known pathogenic human mtDNA mutations are located in the tRNA genes, although these genes only occupy 9.1% of the genome [42], implying that these changes are, at low levels of heteroplasmy, more compatible with life than some protein-coding mutations; and (3) disease-causing tRNA gene mutations reach high heteroplasmic levels, or sometimes must be homoplasmic, before the onset of disease [43,44]. A less acute, but equally important, mechanism of purifying selection appears to be acting on tRNA genes. This may explain why the corresponding mutations are not removed as rapidly from the mtDNA mutator mouse lines.

A very low mutation rate was observed for the control region of mtDNA mutator strains (Figure 4A), despite the fact that the control region sequences are normally the most variable regions in mtDNAs. A reduction in the number of mutations within the control region was also reported in the somatic tissues of the mtDNA mutator mice [38], though a mechanism to explain this observation is still elusive.

## Discussion

We present experimental evidence for strong purifying selection against nonsynonymous mutations in protein-coding genes during maternal transmission of mutated mtDNA in the mouse. The drastic reduction of mutations in the amino acid changing first and second codon positions of protein-coding genes are a direct result of purifying selection against deleterious mtDNA mutations at some stage within the reproductive cycle of these mice. This bias occurs rapidly and is evident as early as the N2 generation. These findings have profound implications for our understanding of how mutated mtDNA is transmitted between generations. It is important to recognize that this strong purifying selection against nonsynonymous changes that we observe is likely to be a universal phenomenon in mammals, but the rapid nature of this selective force would render these mutations difficult to detect in population studies. These findings have profound implications for our understanding of how mutated mtDNA is transmitted between generations.

Within studies of human mtDNA evolution, the observation is that many substitutions are not ancient changes shared deep within human haplogroups, but rather are new variants clustered within the tips of phylogenetic networks and found only in a small number of individuals. This implies they are mildly deleterious variants not yet selected against [23,26,30]. Studies of disease-causing mtDNA mutations show they are often heteroplasmic, and can be present at high levels without consequence for the carrier. However, once the levels exceed a specific threshold, the respiratory chain function will be impaired, causing a clinical phenotype [11]. Based on these observations, when using the mtDNA mutator mouse to study germ line transmission of mtDNA mutations, one could expect to observe the inheritance of high numbers of mutations at all sites in the early generations, which would eventually be removed from the mouse lines once their phenotypic thresholds had been crossed. Whereas the inheritance of the tRNA, rRNA, and third codon position mutations appear to be following this expected pattern (see Figures 2 and 4), this is not the behaviour of mutations at the nonsynonymous first and second codon positions in our mouse lines (Figure 2).

The strongest signature of purifying selection can be observed within *mt-CO1* and *mt-CO2*, consistent with the very high levels of sequence conservation in these genes. The strength and speed of this purifying selection could have other effects on the mutation patterns observed in our model. The consensus view is that bi-parental recombination of mammalian mtDNA is at most extremely rare [3–6] and therefore selection acting at any one site in the mtDNA will affect the entire mtDNA molecule. The observed strong and rapid selection of mtDNA mutations could therefore also reduce the number of neutral variants observed, due to their linkage to deleterious mutations. This means that 4-fold degenerate sites or even noncoding mutations might not be the reliable measure of the mitochondrial neutral mutation rate. Such an underestimation of the mtDNA mutation rate using phylogenetic or population methods relative to pedigree-based observation has been reported previously [45–47]. If this is the case, the models based on this assumption require recalibration.

This point is also important in interpreting the excess change observed for the *mt-CYB*, *mt-ATP6*, and *mt-ATP8* genes in mtDNA mutator lines. Similar gene-specific increases of mutations have been reported in human mtDNA, especially in *mt-ATP6* [23–25,28,29]. Though some argue that this signifies positive selection, the pattern may also be due to less-intense purifying selection on these specific genes. If mutations at *mt-ATP6* experience less-selective constraint, mutations at these sites will be allowed to accumulate and persist in the mtDNA pool. Meanwhile, mutations at strongly selected sites, such as *mt-CO1* and *mt-CO2*, are eliminated, leading to the relative increase in the observed frequency of *mt-ATP6* and *mt-ATP8* mutations in our model organisms.

In contrast to the rapid selection against nonsynonymous changes, rRNA and tRNA genes experienced less-intense purifying selection in our mtDNA mutator lines. Though tRNA genes also have high levels of sequence conservation, the frequency of observed mutations at these sites in our mouse lines was quite similar to the rate observed at third codon positions (Figures 2A and 4A). Some of the identified tRNA mutations, e.g., the deletion of one base in the anticodon loop of *mt-TM* (3873delC mutation) can be predicted to have a biochemical effect if present at high levels. Previous models have mainly been based on observational studies of transmission of mutated mtDNA in human pedigrees affected by mitochondrial disease. Such threshold-mediated protection from selection should lead to slower purifying selection of the mtDNA variant, which may be reflected in the essentially neutral segregation patterns observed for disease-causing mutations prior to clinical manifestation [11,17,27,48].

It is plausible that these tRNAs, as well as a number of the nonsynonymous changes in the protein-coding genes in our model system, may eventually behave like human mtDNA disease mutations in that these mutations are transmitted and cause no obvious phenotype at low levels, but may be selected against or cause a disease-like phenotype at higher levels. We will continue sampling our lines to investigate the long-term fate of the observed tRNA gene mutations, as well as the stably transmitted nonsynonymous protein-coding gene changes.

In the mtDNA mutator mice, the mutations within protein-coding genes are equally distributed across all three codon positions [38,49], whereas the pattern of mutation accumulation is different in the mtDNA mutator lines. It has previously been proposed that mitochondrial fitness may be selected for during oocyte development [50], and it is therefore quite possible that mtDNA in germ cells is under a different selective regime than the mtDNA in somatic cells. There is a massive proliferation of mtDNA during oogenesis, whereby a small number of mtDNA copies in the primordial germ cells are extensively amplified to generate the approximately 10^5^ mtDNA copies in the mature oocyte [2,50]. This mechanism provides ample opportunities for functional testing of mtDNA during female germ-cell development, and future research is required to unravel molecular mechanisms responsible for this selection.

Our experimental strategy has allowed us to look at the fate of a broad spectrum of mtDNA variation, and we report evidence of strong purifying selection in the mouse female germ line. All of the generated mtDNA mutator mouse lines showed the same strong reduction in nonsynonymous substitutions, exemplified by the reduction in first and second codon position mutations. This pattern is also seen in human populations and implies that purifying selection has a similar, drastic impact on the mtDNA variation in humans despite different demographic histories. The RNA genes, in contrast, appear to accumulate at levels approximating the synonymous third codon positions. These mutations are expected to eventually raise to high-enough levels and lead to impaired mitochondrial function in a manner similar to the threshold effect seen in human mtDNA disease. The data generated from this experimental model will allow us to build more accurate molecular models of mtDNA evolution and aid the understanding of inheritance patterns of human mtDNA disease mutations.

## Materials and Methods

### Mouse strains and generation of mouse lines.

Heterozygous knock-in male mice (*PolgA^mut^/+*) [38] were crossed to C57Bl/6NCrl females (Charles River Laboratories). Resulting heterozygous mice were intercrossed to obtain female homozygous knock-in mice (*PolgA^mut^/PolgA^mut^*). We performed crosses of mtDNA mutator females to wild type C57Bl/6 males to produce N1 females. Maternal mouse lines were then established by successive backcrossing of females to C57Bl/6 males (Figure 1). The genotype at the *PolgA* locus was determined as described previously [38], and from the N2 generation onwards, only mice homozygous for the wild-type *PolgA-*allele were used in the study. In two cases, we bred heterozygous knock-in females from the N2 generation because of small litter sizes. In these two lines, all animals were homozygous for the wild-type *PolgA-*allele from generation N3 and onwards.

This animal study was approved by the animal welfare ethics committee and performed in compliance with Swedish law.

### DNA amplification and sequencing.

Pups were weaned at 21–25 d, and tissue from an ear punch was used for DNA isolation, as previously described [51], except that the DNA was purified by phenol:chloroform:isoamyl alcohol (25:24:1) and chloroform extraction, followed by sodium acetate salt and ethanol precipitation. DNA was dissolved in an appropriate volume of deionised water.

The mtDNA genome of each animal was amplified in 29 overlapping PCR reactions (Table S4). All PCR primers contain 5′ M13F or M13R sequence to use as sequencing primers. PCR samples were cleaned using the Agencourt AMPure PCR purification and directly sequenced in both directions using BigDye version 3.1 sequencing kit (ABI). Cycle sequencing reactions were cleaned by precipitation (ABI protocol) or by the Agencourt CleanSEQ Dye-terminator removal kit.

### Data analysis.

The sequence reactions were analysed on a 3130xl capillary sequencer (ABI), and assembled and analysed for heteroplasmies and substitutions using SeqScape V2.5 software (ABI) and compared to our C57Bl/6 mtDNA reference sequence. The software identified heteroplasmy or substitutions at ≥25% signal intensity on both strands of DNA sequence. All heteroplasmies were confirmed by eye. All mutation sites are potentially heteroplasmic in this analysis, due to these detection thresholds in sequencing technology [52]. The wild-type C57Bl/6 mtDNA sequence used in this study varied from that presented in GenBank (http://www.ncbi.nlm.nih.gov/Genbank) accession number NC_005089.1 in two sites; position 9,461 was C (synonymous change at amino acid 1 of *mt-ND3*) and position 11,515 was A (S450N, nonsynonymous change in *mt-ND4*). Two poly-A tracts in the mouse genome could not be reliably scored for insertion and deletions due to sequencing complications. We therefore ignored insertions/deletions at positions 5,160–5,191 (origin of light strand replication) and 9,821–9,830 (polymorphic region of *mt-tR*).

The following mouse sequences were used for comparison of strain variation, GenBank accession numbers: AB042432, AB042523, AB042524, AB042809, AB049357, AJ489607, AJ512208, AY339599, AY466499, AY533105, AY533106, AY533107, AY533108, AY675564, AY999076, DQ106412, DQ106413, J01420, L07095, L07096, and V00711. Sequences were aligned to the wild-type C57Bl/6 mtDNA sequence, and all variations were scored.

Human mtDNA sequence data were obtained from the mtDB human database (http://www.genpat.uu.se/mtDB/; accessed February 2007) [16]. Variants were classified as all observed sequence changes from the most prominently observed nucleotide at the given position in the database.

Codon usage for our C57Bl/6 line was calculated using CodonW version 1.3 (John Peden, http://codonw.sourceforge.net//). The codon usage was used to calculate the number of synonymous and nonsynonymous substitution sites in the C57Bl/6 mtDNA genome.

The number of 4-fold degenerate sites versus other sites for the protein-coding genes was derived from codon usage calculations on each protein-coding gene. Expect values (Figure 3A and 3B) made the assumption of a random distribution of observed mutations across the mtDNA molecule. The genome total of observed mutations within each class was multiplied by the proportion of those sites encoded for each gene. Observed mutations are reported as the number of mutations for that gene detected, multiplied by the proportion of sites for that class within that gene.

### Statistical tests.

Comparison of nucleotide biases was conducted using a contingency analysis on the data supplied in Table 1. The analysis was performed on the eight substitution classes, where all observed values were greater than six. The variation was observed to be significant (chi-square test, *p* = 0.0023, 7 *df*).

Comparisons of the 4-fold versus non–4-fold mutations for each mitochondrial OXPHOS enzyme complex were carried out using the Fisher exact test on a contingency table comparing each enzyme subunit to the sum of mutations for the remaining complexes. The Bonferroni correction for multiple testing of the data was applied when determining significance.

## Supporting Information

Dataset S1 Compilation of Observed Mutations in mtDNA Mutator Mouse Lines (1.2 MB DOC)

Figure S1 Plot of mtDNA Protein-Coding Mutations Observed by Codon Position in the N2 Generation The mutations per base pair observed in N2 animals are plotted by codon position and divided into synonymous (open bars) and nonsynonymous (filled bars) substitutions. A total of 343 mutations were observed: 90 in the first codon position (19 synonymous and 71 nonsynonymous), 70 in the second, and 183 in the third (165 synonymous and 18 nonsynonymous).
(1.1 MB AI).

Table S1 McDonald-Kreitman Test of Neutrality Conducted on the mtDNA Mutator Mouse Lines Values calculated for the McDonald-Kreitman (MK) test of neutrality [39] and the accompanying Neutrality Index (NI) [40], comparing the mtDNA mutator line dataset to the mtDNA sequences of *M. m. molossinus* and *M. m. domesticus* NZB mouse strains. The mtDNA mutator lines show statistically significant excess of mtDNA nonsynonymous mutations when compared to either outgroup (*p-*values of a 2 × 2 contingency table using the Fisher exact test are reported). Values are also provided when comparing *M. m. molossinus* mtDNA to the NZB and C57Bl/6 mtDNA sequences (also statistically significant excess in nonsynonymous substitutions).
(30 KB DOC)

Table S2 Observed Mutations in Mouse Lines, by Class Data accompanying Figures 2A and 4A. These values were divided by the number of nucleotides represented by (A) protein-coding genes divided into synonymous and nonsynonymous mutations and (B) tRNAs, rRNAs, and the control region.
(32 KB DOC)

Table S3 Mutation Occurrence at 4-Fold Degenerate Sites and All Other Protein-Coding Sites, by Gene Data accompanying Figure 3. The expected values were calculated as a proportion of the total observed hits, equally distributed across the number of sites per gene for the two data classes for (A) mtDNA mutator mouse lines and (B) the human mtDB dataset.
(73 KB DOC)

Table S4 Primers Used for Mouse mtDNA Amplification and Sequencing (89 KB DOC)

### Accession Numbers

The GenBank (http://www.ncbi.nlm.nih.gov/Genbank) accession numbers for the mouse sequences used for comparison of strain variation to the wild-type C57Bl/6 mtDNA sequence (DQ106412) are as follows: AB042432, AB042523, AB042524, AB042809, AB049357, AJ489607, AJ512208, AY339599, AY466499, AY533105, AY533106, AY533107, AY533108, AY675564, AY999076, DQ106413, J01420, L07095, L07096, and V00711.

## Abbreviations

mtDNA: mitochondrial DNA
OXPHOS: oxidative phosphorylation
