# Supplementary material for: Strong Purifying Selection in Transmission of Mammalian Mitochondrial DNA
Source: PLoS Biol. 2008 Jan 29;6(1):e10. doi: 10.1371/journal.pbio.0060010 (PMC2214808; doi:10.1371/journal.pbio.0060010)
Supplement: Dataset S1 — (1.2 MB DOC) [file pbio.0060010.sd001.doc]

**Data Set 1.** Compilation of observed mutations in mtDNA mutator mouse lines.

| **Genome Position** | **Gene** | **nucleotide change** | **amino acid change** |
| --- | --- | --- | --- |
| 5 | *mt-TF* | a>T |  |
| 10 | *mt-TF* | g>A |  |
| 23 | *mt-TF* | g>A |  |
| 29 | *mt-TF* | c>T |  |
| 43 | *mt-TF* | a>T |  |
| 52 | *mt-TF* | a>G |  |
| 56 | *mt-TF* | t>A |  |
| 59 | *mt-TF* | c>T |  |
| 69 | *mt-RNR1* | c>T |  |
| 80 | *mt-RNR1* | t>A |  |
| 96 | *mt-RNR1* | a>T |  |
| 105 | *mt-RNR1* | t>C |  |
| 113 | *mt-RNR1* | c>G |  |
| 120 | *mt-RNR1* | a>G |  |
| 140 | *mt-RNR1* | a>T |  |
| 153 | *mt-RNR1* | del C |  |
| 156 | *mt-RNR1* | t>G |  |
| 158 | *mt-RNR1* | a>G |  |
| 172 | *mt-RNR1* | g>A |  |
| 173 | *mt-RNR1* | a>G |  |
| 180 | *mt-RNR1* | a>G |  |
| 182 | *mt-RNR1* | c>T |  |
| 187 | *mt-RNR1* | a>G |  |
| 189 | *mt-RNR1* | a>T |  |
| 190 | *mt-RNR1* | t>C |  |
| 213 | *mt-RNR1* | c>G |  |
| 222 | *mt-RNR1* | a>T |  |
| 228 | *mt-RNR1* | a>G |  |
| 232 | *mt-RNR1* | g>A |  |
| 244 | *mt-RNR1* | a>G |  |
| 255 | *mt-RNR1* | g>A |  |
| 282 | *mt-RNR1* | t>A |  |
| 287 | *mt-RNR1* | c>T |  |
| 322 | *mt-RNR1* | t>A |  |
| 336 | *mt-RNR1* | c>T |  |
| 354 | *mt-RNR1* | c>G |  |
| 363 | *mt-RNR1* | t>G |  |
| 364 | *mt-RNR1* | g>A |  |
| 366 | *mt-RNR1* | c>T |  |
| 368 | *mt-RNR1* | a>G |  |
| 378 | *mt-RNR1* | a>G |  |
| 384 | *mt-RNR1* | t>A |  |
| 386 | *mt-RNR1* | g>A |  |
| 426 | *mt-RNR1* | a>T |  |
| 452 | *mt-RNR1* | c>T |  |
| 454 | *mt-RNR1* | a>G |  |
| 466 | *mt-RNR1* | t>G |  |
| 466 | *mt-RNR1* | t>C |  |
| 478 | *mt-RNR1* | a>T |  |
| 480 | *mt-RNR1* | g>A |  |
| 500 | *mt-RNR1* | a>G |  |
| 512 | *mt-RNR1* | t>C |  |
| 535 | *mt-RNR1* | a>G |  |
| 549 | *mt-RNR1* | a>T |  |
| 552 | *mt-RNR1* | t>G |  |
| 554 | *mt-RNR1* | c>G |  |
| 603 | *mt-RNR1* | t>C |  |
| 604 | *mt-RNR1* | del T |  |
| 606 | *mt-RNR1* | t>C |  |
| 632 | *mt-RNR1* | a>G |  |
| 641 | *mt-RNR1* | a>T |  |
| 649 | *mt-RNR1* | c>T |  |
| 656 | *mt-RNR1* | a>G |  |
| 667 | *mt-RNR1* | del C |  |
| 699 | *mt-RNR1* | a>G |  |
| 712 | *mt-RNR1* | t>A |  |
| 718 | *mt-RNR1* | del A |  |
| 721 | *mt-RNR1* | a>T |  |
| 731 | *mt-RNR1* | a>G |  |
| 740 | *mt-RNR1* | a>G |  |
| 758 | *mt-RNR1* | t>C |  |
| 758 | *mt-RNR1* | t>G |  |
| 760 | *mt-RNR1* | t>C |  |
| 762 | *mt-RNR1* | g>C |  |
| 767 | *mt-RNR1* | t>C |  |
| 782 | *mt-RNR1* | t>A |  |
| 795 | *mt-RNR1* | c>T |  |
| 812 | *mt-RNR1* | c>T |  |
| 813 | *mt-RNR1* | t>C |  |
| 816 | *mt-RNR1* | a>T |  |
| 833 | *mt-RNR1* | a>G |  |
| 858 | *mt-RNR1* | a>G |  |
| 873 | *mt-RNR1* | c>T |  |
| 887 | *mt-RNR1* | g>C |  |
| 898 | *mt-RNR1* | c>T |  |
| 901 | *mt-RNR1* | a>T |  |
| 917 | *mt-RNR1* | c>T |  |
| 934 | *mt-RNR1* | a>T |  |
| 946 | *mt-RNR1* | del A |  |
| 949 | *mt-RNR1* | a>G |  |
| 952 | *mt-RNR1* | t>C |  |
| 953 | *mt-RNR1* | t>C |  |
| 958 | *mt-RNR1* | a>G |  |
| 1025 | *mt-TV* | c>T |  |
| 1027 | *mt-TV* | t>C |  |
| 1030 | *mt-TV* | t>G |  |
| 1031 | *mt-TV* | g>A |  |
| 1073 | *mt-TV* | c>T |  |
| 1078 | *mt-TV* | c>T |  |
| 1084 | *mt-TV* | a>G |  |
| 1086 | *mt-TV* | c>T |  |
| 1091 | *mt-TV* | t>C |  |
| 1109 | *mt-RNR2* | a>G |  |
| 1128 | *mt-RNR2* | t>C |  |
| 1135 | *mt-RNR2* | t>C |  |
| 1160 | *mt-RNR2* | a>T |  |
| 1185 | *mt-RNR2* | c>T |  |
| 1188 | *mt-RNR2* | a>G |  |
| 1191 | *mt-RNR2* | t>C |  |
| 1211 | *mt-RNR2* | t>A |  |
| 1224 | *mt-RNR2* | a>G |  |
| 1230 | *mt-RNR2* | a>G |  |
| 1251 | *mt-RNR2* | c>T |  |
| 1276 | *mt-RNR2* | t>C |  |
| 1283 | *mt-RNR2* | a>G |  |
| 1288 | *mt-RNR2* | a>G |  |
| 1300 | *mt-RNR2* | a>G |  |
| 1300 | *mt-RNR2* | a>T |  |
| 1304 | *mt-RNR2* | c>T |  |
| 1309 | *mt-RNR2* | t>C |  |
| 1314 | *mt-RNR2* | g>A |  |
| 1323 | *mt-RNR2* | c>T |  |
| 1328 | *mt-RNR2* | a>G |  |
| 1354 | *mt-RNR2* | a>T |  |
| 1357 | *mt-RNR2* | a>C |  |
| 1377 | *mt-RNR2* | g>A |  |
| 1378 | *mt-RNR2* | t>C |  |
| 1379 | *mt-RNR2* | c>T |  |
| 1381 | *mt-RNR2* | a>G |  |
| 1398 | *mt-RNR2* | g>A |  |
| 1399 | *mt-RNR2* | a>G |  |
| 1420 | *mt-RNR2* | a>C |  |
| 1441 | *mt-RNR2* | t>C |  |
| 1445 | *mt-RNR2* | t>C |  |
| 1458 | *mt-RNR2* | a>G |  |
| 1467 | *mt-RNR2* | a>T |  |
| 1475 | *mt-RNR2* | t>C |  |
| 1506 | *mt-RNR2* | a>C |  |
| 1511 | *mt-RNR2* | t>C |  |
| 1512 | *mt-RNR2* | a>T |  |
| 1544 | *mt-RNR2* | c>T |  |
| 1547 | *mt-RNR2* | c>T |  |
| 1558 | *mt-RNR2* | a>G |  |
| 1559 | *mt-RNR2* | a>G |  |
| 1611 | *mt-RNR2* | del A |  |
| 1612 | *mt-RNR2* | a>G |  |
| 1619 | *mt-RNR2* | c>T |  |
| 1679 | *mt-RNR2* | c>T |  |
| 1705 | *mt-RNR2* | t>C |  |
| 1728 | *mt-RNR2* | a>G |  |
| 1746 | *mt-RNR2* | a>T |  |
| 1775 | *mt-RNR2* | g>A |  |
| 1779 | *mt-RNR2* | a>G |  |
| 1805 | *mt-RNR2* | a>T |  |
| 1808 | *mt-RNR2* | c>T |  |
| 1820 | *mt-RNR2* | a>C |  |
| 1824 | *mt-RNR2* | a>C |  |
| 1840 | *mt-RNR2* | t>C |  |
| 1841 | *mt-RNR2* | c>T |  |
| 1852 | *mt-RNR2* | t>C |  |
| 1876 | *mt-RNR2* | t>G |  |
| 1895 | *mt-RNR2* | a>T |  |
| 1907 | *mt-RNR2* | g>A |  |
| 1935 | *mt-RNR2* | t>C |  |
| 1944 | *mt-RNR2* | a>T |  |
| 1976 | *mt-RNR2* | a>T |  |
| 1994 | *mt-RNR2* | a>T |  |
| 2001 | *mt-RNR2* | a>T |  |
| 2003 | *mt-RNR2* | c>T |  |
| 2026 | *mt-RNR2* | a>T |  |
| 2033 | *mt-RNR2* | g>A |  |
| 2039 | *mt-RNR2* | t>C |  |
| 2040 | *mt-RNR2* | c>T |  |
| 2048 | *mt-RNR2* | c>T |  |
| 2054 | *mt-RNR2* | t>C |  |
| 2061 | *mt-RNR2* | c>T |  |
| 2086 | *mt-RNR2* | c>T |  |
| 2088 | *mt-RNR2* | a>G |  |
| 2094 | *mt-RNR2* | c>T |  |
| 2096 | *mt-RNR2* | c>T |  |
| 2188 | *mt-RNR2* | t>A |  |
| 2243 | *mt-RNR2* | a>G |  |
| 2250 | *mt-RNR2* | t>C |  |
| 2256 | *mt-RNR2* | t>A |  |
| 2266 | *mt-RNR2* | g>A |  |
| 2281 | *mt-RNR2* | c>T |  |
| 2284 | *mt-RNR2* | a>G |  |
| 2298 | *mt-RNR2* | g>A |  |
| 2323 | *mt-RNR2* | c>T |  |
| 2340 | *mt-RNR2* | g>A |  |
| 2392 | *mt-RNR2* | a>T |  |
| 2398 | *mt-RNR2* | g>A |  |
| 2441 | *mt-RNR2* | c>T |  |
| 2463 | *mt-RNR2* | a>G |  |
| 2467 | *mt-RNR2* | a>T |  |
| 2475 | *mt-RNR2* | t>C |  |
| 2493 | *mt-RNR2* | a>T |  |
| 2560 | *mt-RNR2* | c>T |  |
| 2585 | *mt-RNR2* | a>T |  |
| 2591 | *mt-RNR2* | a>C |  |
| 2605 | *mt-RNR2* | c>T |  |
| 2631 | *mt-RNR2* | a>G |  |
| 2645 | *mt-RNR2* | t>C |  |
| 2648 | *mt-RNR2* | a>T |  |
| 2652 | *mt-RNR2* | a>T |  |
| 2655 | *mt-RNR2* | c>T |  |
| 2656 | *mt-RNR2* | c>T |  |
| 2658 | *mt-RNR2* | t>G |  |
| 2659 | *mt-RNR2* | c>T |  |
| 2679 | *mt-RNR2* | a>G |  |
| 2685 | *mt-TL1* | g>A |  |
| 2761 | *mt-ND1* | t>C | I>T |
| 2765 | *mt-ND1* | t>C | N>N |
| 2780 | *mt-ND1* | c>T | L>L |
| 2787 | *mt-ND1* | a>G | I>V |
| 2790 | *mt-ND1* | c>T | L>L |
| 2795 | *mt-ND1* | c>T | I>I |
| 2798 | *mt-ND1* | c>T | A>A |
| 2825 | *mt-ND1* | c>T | R>R |
| 2843 | *mt-ND1* | a>T | M>I |
| 2864 | *mt-ND1* | c>A | N>K |
| 2869 | *mt-ND1* | t>C | V>A |
| 2875 | *mt-ND1* | c>T | P>L |
| 2888 | *mt-ND1* | a>T | L>F |
| 2899 | *mt-ND1* | c>T | A>V |
| 2901 | *mt-ND1* | g>A | D>N |
| 2903 | *mt-ND1* | c>T | D>D |
| 2925 | *mt-ND1* | g>A | E>K |
| 2926 | *mt-ND1* | a>G | E>G |
| 2930 | *mt-ND1* | a>G | P>P |
| 2931 | *mt-ND1* | a>G | M>V |
| 2944 | *mt-ND1* | c>T | T>M |
| 2981 | *mt-ND1* | a>T | L>L |
| 2990 | *mt-ND1* | a>T | T>T |
| 3014 | *mt-ND1* | c>T | P>P |
| 3017 | *mt-ND1* | a>T | L>L |
| 3023 | *mt-ND1* | a>T | M>I |
| 3073 | *mt-ND1* | c>T | T>M |
| 3129 | *mt-ND1* | t>A | Y>N |
| 3138 | *mt-ND1* | t>C | F>L |
| 3158 | *mt-ND1* | a>T | V>V |
| 3184 | *mt-ND1* | c>T | T>I |
| 3192 | *mt-ND1* | a>G | I>V |
| 3214 | *mt-ND1* | t>A | L>TERM |
| 3224 | *mt-ND1* | a>G | G>G |
| 3245 | *mt-ND1* | t>C | L>L |
| 3258 | *mt-ND1* | g>A | E>K |
| 3272 | *mt-ND1* | a>G | L>L |
| 3315 | *mt-ND1* | a>G | T>A |
| 3323 | *mt-ND1* | a>G | A>A |
| 3324 | *mt-ND1* | g>A | E>K |
| 3358 | *mt-ND1* | g>A | G>D |
| 3368 | *mt-ND1* | a>G | E>E |
| 3375 | *mt-ND1* | t>C | S>P |
| 3411 | *mt-ND1* | g>A | A>T |
| 3434 | *mt-ND1* | c>T | Y>Y |
| 3455 | *mt-ND1* | c>T | N>N |
| 3496 | *mt-ND1* | t>C | I>T |
| 3509 | *mt-ND1* | a>G | E>E |
| 3535 | *mt-ND1* | a>G | E>G |
| 3538 | *mt-ND1* | c>T | A>V |
| 3542 | *mt-ND1* | a>T | L>L |
| 3545 | *mt-ND1* | a>T | L>L |
| 3553 | *mt-ND1* | c>T | S>L |
| 3555 | *mt-ND1* | a>T | T>S |
| 3574 | *mt-ND1* | c>T | A>V |
| 3581 | *mt-ND1* | t>C | Y>Y |
| 3582 | *mt-ND1* | c>T | P>S |
| 3584 | *mt-ND1* | a>T | P>P |
| 3615 | *mt-ND1* | c>T | L>L |
| 3628 | *mt-ND1* | t>C | F>S |
| 3630 | *mt-ND1* | c>T | L>L |
| 3641 | *mt-ND1* | a>G | T>T |
| 3646 | *mt-ND1* | c>T | A>V |
| 3662 | *mt-ND1* | t>C | H>H |
| 3671 | *mt-ND1* | a>T | L>F |
| 3679 | *mt-ND1* | t>C | F>S |
| 3685 | *mt-ND1* | c>T | A>V |
| 3710 | *mt-TI* | a>G |  |
| 3746 | *mt-TI* | t>C |  |
| 3754 | *mt-TI* | g>A |  |
| 3757 | *mt-TI* | c>T |  |
| 3760 | *mt-TI* | g>A |  |
| 3805 | *mt-TQ* | a>G |  |
| 3869 | *mt-TM* | c>T |  |
| 3873 | *mt-TM* | del C |  |
| 3873 | *mt-TM* | c>G |  |
| 3874 | *mt-TM* | c>T |  |
| 3887 | *mt-TM* | a>G |  |
| 3897 | *mt-TM* | a>T |  |
| 3898 | *mt-TM* | a>G |  |
| 3906 | *mt-TM* | c>T |  |
| 3913 | *mt-TM* | a>T |  |
| 3915 | *mt-ND2* | t>A | M>K |
| 3920 | *mt-ND2* | c>T | P>L |
| 3937 | *mt-ND2* | c>T | i>i |
| 3940 | *mt-ND2* | c>T | I>I |
| 3948 | *mt-ND2* | c>T | T>M |
| 3952 | *mt-ND2* | c>T | I>I |
| 3955 | *mt-ND2* | c>T | F>F |
| 3958 | *mt-ND2* | a>G | L>L |
| 3975 | *mt-ND2* | t>C | M>T |
| 3985 | *mt-ND2* | c>T | T>T |
| 4003 | *mt-ND2* | a>G | W>W |
| 4015 | *mt-ND2* | a>G | E>E |
| 4021 | *mt-ND2* | c>T | S>S |
| 4045 | *mt-ND2* | a>G | L>L |
| 4060 | *mt-ND2* | c>T | N>N |
| 4083 | *mt-ND2* | c>T | T>M |
| 4090 | *mt-ND2* | c>T | Y>Y |
| 4099 | *mt-ND2* | a>T | T>T |
| 4102 | *mt-ND2* | a>G | Q>Q |
| 4104 | *mt-ND2* | c>T | A>V |
| 4107 | *mt-ND2* | c>T | T>M |
| 4135 | *mt-ND2* | c>T | I>I |
| 4171 | *mt-ND2* | t>C | F>F |
| 4186 | *mt-ND2* | c>T | N>N |
| 4190 | *mt-ND2* | c>T | L>L |
| 4208 | *mt-ND2* | t>C | L>L |
| 4219 | *mt-ND2* | a>G | L>L |
| 4228 | *mt-ND2* | a>G | K>K |
| 4381 | *mt-ND2* | a>T | M>I |
| 4382 | *mt-ND2* | c>T | L>L |
| 4488 | *mt-ND2* | c>T | A>V |
| 4499 | *mt-ND2* | t>C | Y>H |
| 4501 | *mt-ND2* | c>T | Y>Y |
| 4509 | *mt-ND2* | c>T | S>F |
| 4534 | *mt-ND2* | c>T | I>I |
| 4535 | *mt-ND2* | t>C | Y>H |
| 4556 | *mt-ND2* | a>G | M>V |
| 4577 | *mt-ND2* | a>T | N>Y |
| 4587 | *mt-ND2* | t>C | M>T |
| 4593 | *mt-ND2* | t>C | I>T |
| 4598 | *mt-ND2* | t>C | S>P |
| 4599 | *mt-ND2* | c>T | S>L |
| 4600 | *mt-ND2* | a>G | S>S |
| 4610 | *mt-ND2* | c>T | L>L |
| 4630 | *mt-ND2* | a>G | A>A |
| 4634 | *mt-ND2* | c>T | L>L |
| 4645 | *mt-ND2* | c>T | I>I |
| 4656 | *mt-ND2* | t>C | L>S |
| 4698 | *mt-ND2* | c>T | P>L |
| 4709 | *mt-ND2* | a>G | I>V |
| 4729 | *mt-ND2* | a>G | K>K |
| 4746 | *mt-ND2* | t>A | M>K |
| 4751 | *mt-ND2* | a>G | T>A |
| 4752 | *mt-ND2* | c>T | T>M |
| 4756 | *mt-ND2* | c>T | L>L |
| 4786 | *mt-ND2* | c>T | F>F |
| 4815 | *mt-ND2* | c>T | S>L |
| 4860 | *mt-ND2* | a>G | H>R |
| 4873 | *mt-ND2* | t>G | T>T |
| 4896 | *mt-ND2* | c>T | T>I |
| 4897 | *mt-ND2* | c>T | T>T |
| 4907 | *mt-ND2* | a>G | M>T |
| 4909 | *mt-ND2* | a>T | M>I |
| 4918 | *mt-ND2* | a>T | M>I |
| 4930 | *mt-ND2* | a>G | L>L |
| 4932 | *mt-ND2* | c>T | A>V |
| 4939 | *mt-ND2* | a>T | Q>H |
| 4946 | *mt-ND2* | a>T | T>S |
| 4962 | *mt-TW* | t>C |  |
| 4963 | *mt-TW* | a>T |  |
| 4973 | *mt-TW* | g>T |  |
| 4985 | *mt-TW* | a>G |  |
| 4990 | *mt-TW* | t>C |  |
| 4991 | *mt-TW* | a>G |  |
| 4996 | *mt-TW* | a>G |  |
| 4998 | *mt-TW* | c>T |  |
| 5025 | *mt-TA* | t>A |  |
| 5027 | *mt-TA* | t>A |  |
| 5045 | *mt-TA* | c>T |  |
| 5076 | *mt-TA* | g>A |  |
| 5077 | *mt-TA* | c>T |  |
| 5078 | *mt-TA* | t>A |  |
| 5088 | non-coding | a>G |  |
| 5099 | *mt-TN* | a>G |  |
| 5105 | *mt-TN* | t>A |  |
| 5114 | *mt-TN* | g>C |  |
| 5124 | *mt-TN* | t>C |  |
| 5127 | *mt-TN* | a>G |  |
| 5128 | *mt-TN* | c>T |  |
| 5151 | *mt-TN* | t>C |  |
| 5153 | *mt-TN* | c>T |  |
| 5196 | *mt-TC* | c>T |  |
| 5199 | *mt-TC* | a>T |  |
| 5206 | *mt-TC* | a>T |  |
| 5212 | *mt-TC* | c>T |  |
| 5221 | *mt-TC* | c>T |  |
| 5224 | *mt-TC* | a>G |  |
| 5229 | *mt-TC* | c>T |  |
| 5234 | *mt-TC* | c>T |  |
| 5242 | *mt-TC* | a>G |  |
| 5243 | *mt-TC* | t>C |  |
| 5245 | *mt-TC* | t>C |  |
| 5275 | *mt-TY* | t>A |  |
| 5279 | *mt-TY* | a>C |  |
| 5297 | *mt-TY* | a>T |  |
| 5302 | *mt-TY* | c>T |  |
| 5314 | *mt-TY* | c>T |  |
| 5327 | non-coding | t>C |  |
| 5332 | *mt-CO1* | t>C | F>S |
| 5351 | *mt-CO1* | c>T | F>F |
| 5360 | *mt-CO1* | t>C | N>N |
| 5372 | *mt-CO1* | c>T | L>L |
| 5381 | *mt-CO1* | c>T | L>L |
| 5385 | *mt-CO1* | c>T | L>L |
| 5395 | *mt-CO1* | g>T | G>V |
| 5484 | *mt-CO1* | a>G | I>V |
| 5489 | *mt-CO1* | c>T | Y>Y |
| 5495 | *mt-CO1* | t>C | V>V |
| 5499 | *mt-CO1* | g>A | V>M |
| 5528 | *mt-CO1* | c>T | F>F |
| 5576 | *mt-CO1* | c>G | V>V |
| 5630 | *mt-CO1* | t>C | S>S |
| 5714 | *mt-CO1* | c>T | Y>Y |
| 5741 | *mt-CO1* | t>C | H>H |
| 5777 | *mt-CO1* | t>A | L>L |
| 5801 | *mt-CO1* | t>C | I>I |
| 5825 | *mt-CO1* | c>T | T>T |
| 5834 | *mt-CO1* | c>T | I>I |
| 5869 | *mt-CO1* | c>T | T>I |
| 5891 | *mt-CO1* | a>T | V>V |
| 5921 | *mt-CO1* | a>T | S>S |
| 6008 | *mt-CO1* | c>T | D>D |
| 6020 | *mt-CO1* | c>T | Y>Y |
| 6065 | *mt-CO1* | t>C | L>L |
| 6080 | *mt-CO1* | t>A | F>L |
| 6216 | *mt-CO1* | t>C | L>L |
| 6276 | *mt-CO1* | g>T | G>C |
| 6276 | *mt-CO1* | g>A | G>S |
| 6279 | *mt-CO1* | g>A | V>I |
| 6281 | *mt-CO1* | c>T | V>V |
| 6300 | *mt-CO1* | g>A | A>T |
| 6305 | *mt-CO1* | c>T | T>T |
| 6317 | *mt-CO1* | t>C | G>G |
| 6342 | *mt-CO1* | c>T | L>L |
| 6377 | *mt-CO1* | t>A | V>V |
| 6377 | *mt-CO1* | t>C | V>V |
| 6398 | *mt-CO1* | t>C | V>V |
| 6428 | *mt-CO1* | t>C | L>L |
| 6431 | *mt-CO1* | c>T | H>H |
| 6435 | *mt-CO1* | a>T | T>S |
| 6481 | *mt-CO1* | c>T | A>V |
| 6491 | *mt-CO1* | t>C | A>A |
| 6493 | *mt-CO1* | t>C | I>T |
| 6536 | *mt-CO1* | c>T | F>F |
| 6542 | *mt-CO1* | a>G | L>L |
| 6578 | *mt-CO1* | a>G | M>M |
| 6581 | *mt-CO1* | c>T | F>F |
| 6589 | *mt-CO1* | t>C | V>A |
| 6605 | *mt-CO1* | c>T | F>F |
| 6614 | *mt-CO1* | t>C | H>H |
| 6620 | *mt-CO1* | g>A | L>L |
| 6632 | *mt-CO1* | a>G | G>G |
| 6635 | *mt-CO1* | a>G | M>M |
| 6649 | *mt-CO1* | c>T | S>L |
| 6655 | *mt-CO1* | a>T | Y>F |
| 6713 | *mt-CO1* | a>T | Y>Y |
| 6722 | *mt-CO1* | t>C | V>V |
| 6723 | *mt-CO1* | c>T | L>F |
| 6756 | *mt-CO1* | g>A | A>T |
| 6758 | *mt-CO1* | t>C | A>A |
| 6778 | *mt-CO1* | c>T | S>L |
| 6783 | *mt-CO1* | t>C | S>P |
| 6784 | *mt-CO1* | c>T | S>L |
| 6785 | *mt-CO1* | g>A | S>S |
| 6809 | *mt-CO1* | a>T | W>C |
| 6827 | *mt-CO1* | a>G | P>P |
| 6829 | *mt-CO1* | c>T | P>L |
| 6836 | *mt-CO1* | c>T | H>H |
| 6842 | *mt-CO1* | c>T | F>F |
| 6843 | *mt-CO1* | g>A | E>K |
| 6848 | *mt-CO1* | a>T | E>D |
| 6866 | *mt-CO1* | del A | V>del |
| 6868 | *mt-CO1* | a>G | K>TERM |
| 6881 | *mt-TS1* | g>A |  |
| 6886 | *mt-TS1* | c>T |  |
| 6887 | *mt-TS1* | g>A |  |
| 6894 | *mt-TS1* | c>G |  |
| 6902 | *mt-TS1* | g>A |  |
| 6920 | *mt-TS1* | t>A |  |
| 6927 | *mt-TS1* | t>G |  |
| 6931 | *mt-TS1* | t>C |  |
| 6932 | *mt-TS1* | c>T |  |
| 6958 | *mt-TD* | c>T |  |
| 6963 | *mt-TD* | a>G |  |
| 6967 | *mt-TD* | a>G |  |
| 6998 | *mt-TD* | a>G |  |
| 7001 | *mt-TD* | t>A |  |
| 7049 | *mt-CO2* | a>T | T>S |
| 7051 | *mt-CO2* | a>G | T>T |
| 7090 | *mt-CO2* | c>T | H>H |
| 7111 | *mt-CO2* | a>T | L>L |
| 7127 | *mt-CO2* | c>T | L>L |
| 7136 | *mt-CO2* | a>G | I>V |
| 7166 | *mt-CO2* | c>T | H>Y |
| 7185 | *mt-CO2* | c>T | A>V |
| 7214 | *mt-CO2* | c>T | L>L |
| 7225 | *mt-CO2* | a>G | V>V |
| 7228 | *mt-CO2* | c>T | I>I |
| 7243 | *mt-CO2* | t>C | A>A |
| 7255 | *mt-CO2* | a>T | L>L |
| 7291 | *mt-CO2* | c>T | P>P |
| 7292 | *mt-CO2* | g>A | V>A |
| 7300 | *mt-CO2* | c>T | T>T |
| 7308 | *mt-CO2* | c>T | T>I |
| 7310 | *mt-CO2* | a>G | M>V |
| 7312 | *mt-CO2* | a>T | M>I |
| 7317 | *mt-CO2* | a>G | H>R |
| 7336 | *mt-CO2* | c>T | Y>Y |
| 7341 | *mt-CO2* | a>T | Y>F |
| 7350 | *mt-CO2* | a>G | Y>C |
| 7368 | *mt-CO2* | a>G | D>G |
| 7387 | *mt-CO2* | a>G | T>T |
| 7400 | *mt-CO2* | c>T | P>S |
| 7411 | *mt-CO2* | a>G | L>L |
| 7438 | *mt-CO2* | c>T | V>V |
| 7457 | *mt-CO2* | c>T | P>S |
| 7465 | *mt-CO2* | t>A | R>R |
| 7514 | *mt-CO2* | c>T | L>L |
| 7516 | *mt-CO2* | a>G | L>L |
| 7522 | *mt-CO2* | t>A | L>L |
| 7624 | *mt-CO2* | t>C | H>H |
| 7680 | *mt-CO2* | c>T | S>T |
| 7700 | *mt-TK* | c>T |  |
| 7701 | *mt-TK* | a>T |  |
| 7708 | *mt-TK* | a>G |  |
| 7720 | *mt-TK* | t>A |  |
| 7751 | *mt-TK* | del A |  |
| 7754 | *mt-TK* | c>T |  |
| 7758 | *mt-TK* | a>T |  |
| 7838 | *mt-ATP8* | c>T | Q>TERM |
| 7844 | *mt-ATP8* | a>T | K>TERM |
| 7860 | *mt-ATP8* | c>T | T>M |
| 7864 | *mt-ATP8* | c>T | F>F |
| 7867 | *mt-ATP8* | a>T | P>P |
| 7881 | *mt-ATP8* | c>T | P>L |
| 7883 | *mt-ATP8* | a>G | K>E |
| 7884 | *mt-ATP8* | a>G | K>S |
| 7910 | *mt-ATP8* | a>C | T>P |
| 7915 | *mt-ATP8* | t>C | P>P |
| 7924 | *mt-ATP8* | a>T | L>F |
| 7931 | *mt-ATP8* | a>G | T>A |
| 7933 | *mt-ATP8* | g>A | T>T |
| 7949 | *mt-ATP8* | c>T | H>Y |
| 7953 | *mt-ATP8* | c>T | S>L |
| 7964 | *mt-ATP8* | c>T | Q>TERM |
| 7965 | *mt-ATP8* | a>G | T>T |
| 7974 | *mt-ATP8* | a>G | G>G |
| 7984 | *mt-ATP6* | g>A | V>I |
| 8012 | *mt-ATP6* | t>C | I>T |
| 8013 | *mt-ATP6* | c>T | I>I |
| 8045 | *mt-ATP6* | a>G | N>S |
| 8048 | *mt-ATP6* | g>A | R>H |
| 8053 | *mt-ATP6* | c>T | H>Y |
| 8057 | *mt-ATP6* | c>T | S>F |
| 8066 | *mt-ATP6* | a>G | H>R |
| 8088 | *mt-ATP6* | c>T | I>I |
| 8096 | *mt-ATP6* | t>C | M>T |
| 8103 | *mt-ATP6* | a>G | L>L |
| 8106 | *mt-ATP6* | c>T | I>I |
| 8111 | *mt-ATP6* | c>T | T>M |
| 8123 | *mt-ATP6* | g>A | R>Q |
| 8127 | *mt-ATP6* | a>G | T>T |
| 8131 | *mt-ATP6* | a>G | T>A |
| 8145 | *mt-ATP6* | t>C | V>V |
| 8155 | *mt-ATP6* | a>G | M>V |
| 8178 | *mt-ATP6* | c>T | L>L |
| 8181 | *mt-ATP6* | a>T | L>L |
| 8193 | *mt-ATP6* | a>T | P>P |
| 8194 | *mt-ATP6* | c>T | H>Y |
| 8206 | *mt-ATP6* | c>T | P>S |
| 8209 | *mt-ATP6* | a>T | T>S |
| 8239 | *mt-ATP6* | g>A | A>T |
| 8247 | *mt-ATP6* | a>T | P>P |
| 8299 | *mt-ATP6* | c>T | L>L |
| 8307 | *mt-ATP6* | c>T | H>H |
| 8316 | *mt-ATP6* | a>G | P>P |
| 8326 | *mt-ATP6* | c>T | P>S |
| 8359 | *mt-ATP6* | g>A | E>K |
| 8401 | *mt-ATP6* | c>T | R>W |
| 8436 | *mt-ATP6* | a>T | L>F |
| 8448 | *mt-ATP6* | c>T | I>I |
| 8454 | *mt-ATP6* | a>G | G>G |
| 8457 | *mt-ATP6* | t>C | A>A |
| 8469 | *mt-ATP6* | a>T | L>F |
| 8472 | *mt-ATP6* | a>T | M>I |
| 8483 | *mt-ATP6* | c>T | P>L |
| 8514 | *mt-ATP6* | a>G | L>L |
| 8519 | *mt-ATP6* | t>C | L>P |
| 8523 | *mt-ATP6* | c>T | L>L |
| 8546 | *mt-ATP6* | c>T | A>V |
| 8562 | *mt-ATP6* | c>T | Y>Y |
| 8574 | *mt-ATP6* | c>T | L>L |
| 8577 | *mt-ATP6* | a>G | L>L |
| 8589 | *mt-ATP6* | t>C | Y>Y |
| 8602 | *mt-ATP6* | a>T | T>S |
| 8612 | *mt-ATP6* | c>T | T>T |
| 8640 | *mt-CO3* | a>T | N>Y |
| 8665 | *mt-CO3* | g>A | G>E |
| 8670 | *mt-CO3* | t>C | F>L |
| 8678 | *mt-CO3* | c>T | A>A |
| 8685 | *mt-CO3* | c>T | L>L |
| 8691 | *mt-CO3* | t>A | S>T |
| 8692 | *mt-CO3* | c>T | S>L |
| 8693 | *mt-CO3* | a>C | S>S |
| 8704 | *mt-CO3* | t>A | M>K |
| 8714 | *mt-CO3* | c>T | H>H |
| 8727 | *mt-CO3* | a>G | T>A |
| 8747 | *mt-CO3* | a>T | L>L |
| 8755 | *mt-CO3* | a>G | N>S |
| 8804 | *mt-CO3* | c>T | T>T |
| 8807 | *mt-CO3* | c>T | Y>Y |
| 8834 | *mt-CO3* | a>T | Q>H |
| 8835 | *mt-CO3* | a>G | K>E |
| 8843 | *mt-CO3* | a>T | L>L |
| 8870 | *mt-CO3* | c>T | V>V |
| 8878 | *mt-CO3* | t>A | V>E |
| 8924 | *mt-CO3* | c>T | L>L |
| 8968 | *mt-CO3* | t>C | I>T |
| 8969 | *mt-CO3* | t>C | I>I |
| 8971 | *mt-CO3* | c>T | S>L |
| 8992 | *mt-CO3* | t>C | V>A |
| 9046 | *mt-CO3* | c>T | A>V |
| 9052 | *mt-CO3* | a>G | H>R |
| 9053 | *mt-CO3* | t>A | H>Q |
| 9054 | *mt-CO3* | a>T | S>S |
| 9059 | *mt-CO3* | t>C | L>L |
| 9121 | *mt-CO3* | a>T | Y>F |
| 9131 | *mt-CO3* | c>T | I>I |
| 9132 | *mt-CO3* | c>T | L>L |
| 9144 | *mt-CO3* | g>A | E>K |
| 9179 | *mt-CO3* | t>A | G>G |
| 9194 | *mt-CO3* | a>G | T>T |
| 9204 | *mt-CO3* | g>A | A>T |
| 9206 | *mt-CO3* | t>C | A>A |
| 9233 | *mt-CO3* | t>C | I>I |
| 9241 | *mt-CO3* | c>T | S>L |
| 9301 | *mt-CO3* | a>T | H>L |
| 9305 | *mt-CO3* | c>T | F>F |
| 9311 | *mt-CO3* | t>A | F>L |
| 9327 | *mt-CO3* | t>C | Y>H |
| 9336 | *mt-CO3* | t>C | F>L |
| 9341 | *mt-CO3* | a>G | V>V |
| 9344 | *mt-CO3* | c>T | D>D |
| 9362 | *mt-CO3* | a>G | L>L |
| 9364 | *mt-CO3* | a>T | Y>F |
| 9382 | *mt-CO3* | g>A | W>TERM |
| 9392 | *mt-TG* | c>T |  |
| 9399 | *mt-TG* | a>G |  |
| 9401 | *mt-TG* | t>G |  |
| 9410 | *mt-TG* | t>C |  |
| 9419 | *mt-TG* | c>T |  |
| 9442 | *mt-TG* | a>C |  |
| 9444 | *mt-TG* | a>G |  |
| 9463 | *mt-ND3* | a>G | N>S |
| 9482 | *mt-ND3* | c>A | F>L |
| 9500 | *mt-ND3* | c>A | S>S |
| 9501 | *mt-ND3* | c>T | L>L |
| 9503 | *mt-ND3* | a>G | L>L |
| 9505 | *mt-ND3* | c>T | T>M |
| 9507 | *mt-ND3* | c>T | L>L |
| 9522 | *mt-ND3* | t>C | F>L |
| 9547 | *mt-ND3* | a>G | Y>C |
| 9581 | *mt-ND3* | c>T | F>F |
| 9588 | *mt-ND3* | a>T | T>S |
| 9641 | *mt-ND3* | a>T | T>T |
| 9660 | *mt-ND3* | g>A | E>K |
| 9674 | *mt-ND3* | a>T | L>L |
| 9677 | *mt-ND3* | t>G | L>L |
| 9683 | *mt-ND3* | a>G | L>L |
| 9745 | *mt-ND3* | c>T | T>M |
| 9762 | *mt-ND3* | c>A | L>M |
| 9820 | *mt-TR* | t>A |  |
| 9829 | *mt-TR* | t>A |  |
| 9841 | *mt-TR* | a>T |  |
| 9849 | *mt-TR* | g>A |  |
| 9859 | *mt-TR* | a>G |  |
| 9862 | *mt-TR* | t>C |  |
| 9884 | *mt-ND4L* | c>T | S>F |
| 9891 | *mt-ND4L* | c>T | F>F |
| 9920 | *mt-ND4L* | c>T | S>L |
| 9963 | *mt-ND4L* | a>C | T>T |
| 9981 | *mt-ND4L* | c>T | G>G |
| 9988 | *mt-ND4L* | t>C | L>L |
| 10005 | *mt-ND4L* | a>T | M>I |
| 10006 | *mt-ND4L* | a>T | T>S |
| 10014 | *mt-ND4L* | a>G | V>V |
| 10028 | *mt-ND4L* | c>T | S>F |
| 10037 | *mt-ND4L* | t>A | M>K |
| 10056 | *mt-ND4L* | c>T | P>P |
| 10065 | *mt-ND4L* | c>T | I>I |
| 10074 | *mt-ND4L* | c>T | F>F |
| 10099 | *mt-ND4L* | c>T | L>L |
| 10101 | *mt-ND4L* | a>G | L>L |
| 10104 | *mt-ND4L* | c>T | A>A |
| 10130 | *mt-ND4L* | a>G | Y>C |
| 10155 | *mt-ND4L* | c>T | L>L |
| 10185 | *mt-ND4* | c>T | P>S |
| 10190 | *mt-ND4* | a>G | S>S |
| 10199 | *mt-ND4* | a>T | L>L |
| 10217 | *mt-ND4* | a>G | L>L |
| 10230 | *mt-ND4* | a>T | K>Y |
| 10232 | *mt-ND4* | a>T | K>Y |
| 10262 | *mt-ND4* | t>A | F>L |
| 10271 | *mt-ND4* | t>C | S>S |
| 10277 | *mt-ND4* | c>T | T>T |
| 10284 | *mt-ND4* | a>T | T>S |
| 10290 | *mt-ND4* | c>T | L>L |
| 10305 | *mt-ND4* | g>A | E>K |
| 10306 | *mt-ND4* | a>G | E>G |
| 10335 | *mt-ND4* | t>C | S>P |
| 10341 | *mt-ND4* | g>A | D>N |
| 10343 | *mt-ND4* | c>A | D>E |
| 10370 | *mt-ND4* | a>T | L>F |
| 10370 | *mt-ND4* | a>G | L>L |
| 10388 | *mt-ND4* | a>C | P>P |
| 10395 | *mt-ND4* | t>C | L>L |
| 10400 | *mt-ND4* | a>G | M>M |
| 10403 | *mt-ND4* | t>C | A>A |
| 10438 | *mt-ND4* | t>C | L>P |
| 10439 | *mt-ND4* | a>G | L>L |
| 10451 | *mt-ND4* | c>T | Y>Y |
| 10458 | *mt-ND4* | a>G | M>V |
| 10501 | *mt-ND4* | c>T | A>V |
| 10530 | *mt-ND4* | t>G | F>V |
| 10559 | *mt-ND4* | t>A | I>M |
| 10596 | *mt-ND4* | a>T | N>I |
| 10610 | *mt-ND4* | t>C | Y>Y |
| 10611 | *mt-ND4* | t>C | F>L |
| 10624 | *mt-ND4* | c>G | T>M |
| 10632 | *mt-ND4* | g>A | G>S |
| 10637 | *mt-ND4* | t>A | S>S |
| 10715 | *mt-ND4* | a>T | T>T |
| 10716 | *mt-ND4* | c>T | H>Y |
| 10775 | *mt-ND4* | t>C | F>F |
| 10880 | *mt-ND4* | a>G | L>L |
| 10914 | *mt-ND4* | c>T | L>L |
| 10918 | *mt-ND4* | a>C | D>A |
| 10922 | *mt-ND4* | a>G | P>P |
| 10943 | *mt-ND4* | c>T | Y>Y |
| 10961 | *mt-ND4* | c>T | S>S |
| 10974 | *mt-ND4* | a>G | I>V |
| 10981 | *mt-ND4* | c>T | T>I |
| 10995 | *mt-ND4* | t>C | L>L |
| 11016 | *mt-ND4* | t>A | S>T |
| 11018 | *mt-ND4* | a>T | S>S |
| 11034 | *mt-ND4* | t>C | S>P |
| 11041 | *mt-ND4* | g>C | S>T |
| 11045 | *mt-ND4* | c>T | H>H |
| 11085 | *mt-ND4* | t>G | W>G |
| 11102 | *mt-ND4* | a>T | A>A |
| 11108 | *mt-ND4* | a>G | M>M |
| 11115 | *mt-ND4* | a>G | I>V |
| 11123 | *mt-ND4* | t>A | H>H |
| 11129 | *mt-ND4* | c>T | L>L |
| 11150 | *mt-ND4* | c>T | C>C |
| 11153 | *mt-ND4* | a>G | L>L |
| 11168 | *mt-ND4* | c>T | Y>Y |
| 11177 | *mt-ND4* | c>T | I>I |
| 11192 | *mt-ND4* | a>T | M>I |
| 11273 | *mt-ND4* | a>T | L>L |
| 11273 | *mt-ND4* | a>G | L>L |
| 11315 | *mt-ND4* | a>G | M>M |
| 11316 | *mt-ND4* | t>C | S>P |
| 11323 | *mt-ND4* | t>C | F>S |
| 11360 | *mt-ND4* | t>C | I>I |
| 11362 | *mt-ND4* | a>C | N>T |
| 11372 | *mt-ND4* | t>C | I>I |
| 11373 | *mt-ND4* | a>C | T>P |
| 11378 | *mt-ND4* | t>C | G>G |
| 11383 | *mt-ND4* | a>T | Y>F |
| 11391 | *mt-ND4* | t>C | Y>H |
| 11414 | *mt-ND4* | c>G | R>R |
| 11417 | *mt-ND4* | c>T | G>G |
| 11439 | *mt-ND4* | a>T | N>Y |
| 11453 | *mt-ND4* | a>C | S>S |
| 11455 | *mt-ND4* | a>C | H>N |
| 11457 | *mt-ND4* | a>C | T>P |
| 11466 | *mt-ND4* | c>T | L>L |
| 11468 | *mt-ND4* | a>G | L>L |
| 11478 | *mt-ND4* | g>A | A>T |
| 11486 | *mt-ND4* | c>T | H>H |
| 11490 | *mt-ND4* | a>G | I>V |
| 11505 | *mt-ND4* | c>T | L>L |
| 11507 | *mt-ND4* | a>G | L>L |
| 11519 | *mt-ND4* | a>T | P>P |
| 11528 | *mt-ND4* | t>C | I>I |
| 11529 | *mt-ND4* | a>T | T>S |
| 11539 | *mt-ND4* | c>T | T>M |
| 11548 | *mt-TH* | a>T |  |
| 11556 | *mt-TH* | t>G |  |
| 11606 | *mt-TH* | a>G |  |
| 11609 | *mt-TH* | c>T |  |
| 11610 | *mt-TH* | a>G |  |
| 11631 | *mt-TS2* | t>A |  |
| 11666 | *mt-TS2* | t>A |  |
| 11667 | *mt-TS2* | t>C |  |
| 11671 | *mt-TL2* | a>G |  |
| 11689 | *mt-TL2* | t>A |  |
| 11690 | *mt-TL2* | a>G |  |
| 11693 | *mt-TL2* | c>A |  |
| 11694 | *mt-TL2* | c>T |  |
| 11696 | *mt-TL2* | t>C |  |
| 11703 | *mt-TL2* | t>C |  |
| 11722 | *mt-TL2* | t>G |  |
| 11727 | *mt-TL2* | a>G |  |
| 11735 | *mt-TL2* | a>G |  |
| 11755 | *mt-ND5* | c>T | T>M |
| 11804 | *mt-ND5* | t>C | I>I |
| 11826 | *mt-ND5* | c>T | H>Y |
| 11937 | *mt-ND5* | t>G | W>G |
| 11948 | *mt-ND5* | c>T | V>V |
| 11958 | *mt-ND5* | t>C | S>P |
| 11969 | *mt-ND5* | t>A | L>L |
| 11998 | *mt-ND5* | c>T | S>F |
| 12016 | *mt-ND5* | t>G | V>G |
| 12071 | *mt-ND5* | a>T | S>S |
| 12075 | *mt-ND5* | c>T | P>S |
| 12076 | *mt-ND5* | c>T | P>P |
| 12102 | *mt-ND5* | c>T | L>L |
| 12104 | *mt-ND5* | t>C | L>L |
| 12134 | *mt-ND5* | c>T | L>L |
| 12136 | *mt-ND5* | c>T | T>I |
| 12143 | *mt-ND5* | c>T | A>A |
| 12203 | *mt-ND5* | a>G | L>L |
| 12223 | *mt-ND5* | g>A | R>Q |
| 12248 | *mt-ND5* | a>T | L>L |
| 12254 | *mt-ND5* | a>T | A>A |
| 12308 | *mt-ND5* | t>A | F>F |
| 12321 | *mt-ND5* | a>G | N>D |
| 12352 | *mt-ND5* | c>T | S>F |
| 12359 | *mt-ND5* | c>T | N>N |
| 12362 | *mt-ND5* | c>T | N>N |
| 12373 | *mt-ND5* | t>C | I>T |
| 12459 | *mt-ND5* | c>T | P>S |
| 12462 | *mt-ND5* | a>T | T>S |
| 12464 | *mt-ND5* | a>G | T>T |
| 12470 | *mt-ND5* | t>A | V>V |
| 12513 | *mt-ND5* | t>G | F>V |
| 12514 | *mt-ND5* | t>A | F>Y |
| 12530 | *mt-ND5* | c>T | F>F |
| 12531 | *mt-ND5* | c>A | H>N |
| 12533 | *mt-ND5* | c>T | H>H |
| 12546 | *mt-ND5* | a>T | N>Y |
| 12608 | *mt-ND5* | t>C | A>A |
| 12637 | *mt-ND5* | a>T | K>M |
| 12645 | *mt-ND5* | a>G | I>V |
| 12662 | *mt-ND5* | a>T | S>S |
| 12700 | *mt-ND5* | a>G | N>S |
| 12701 | *mt-ND5* | c>T | N>N |
| 12773 | *mt-ND5* | c>G | G>G |
| 12795 | *mt-ND5* | g>A | D>N |
| 12815 | *mt-ND5* | a>G | K>K |
| 12832 | *mt-ND5* | a>T | K>M |
| 12836 | *mt-ND5* | c>T | I>I |
| 12845 | *mt-ND5* | c>T | F>F |
| 12861 | *mt-ND5* | g>A | V>M |
| 12866 | *mt-ND5* | c>T | I>I |
| 12872 | *mt-ND5* | c>T | S>S |
| 12881 | *mt-ND5* | c>T | L>L |
| 12888 | *mt-ND5* | a>G | M>V |
| 12904 | *mt-ND5* | g>A | G>E |
| 12908 | *mt-ND5* | c>T | F>F |
| 12935 | *mt-ND5* | a>T | A>A |
| 12938 | *mt-ND5* | t>C | I>I |
| 12938 | *mt-ND5* | t>A | I>M |
| 12942 | *mt-ND5* | a>T | T>S |
| 12971 | *mt-ND5* | a>G | L>L |
| 12974 | *mt-ND5* | t>C | T>T |
| 12976 | *mt-ND5* | c>T | T>T |
| 12983 | *mt-ND5* | c>T | I>I |
| 13054 | *mt-ND5* | c>T | P>P |
| 13094 | *mt-ND5* | a>T | M>I |
| 13095 | *mt-ND5* | a>G | P>P |
| 13108 | *mt-ND5* | g>A | R>H |
| 13112 | *mt-ND5* | a>G | N>N |
| 13121 | *mt-ND5* | a>G | G>G |
| 13139 | *mt-ND5* | t>A | F>L |
| 13153 | *mt-ND5* | a>G | N>S |
| 13161 | *mt-ND5* | c>T | P>S |
| 13168 | *mt-ND5* | g>A | S>N |
| 13173 | *mt-ND5* | c>T | P>S |
| 13197 | *mt-ND5* | t>C | L>L |
| 13206 | *mt-ND5* | a>T | T>S |
| 13232 | *mt-ND5* | a>G | G>G |
| 13234 | *mt-ND5* | t>A | F>Y |
| 13236 | *mt-ND5* | c>T | L>L |
| 13253 | *mt-ND5* | a>T | E>D |
| 13268 | *mt-ND5* | a>G | M>M |
| 13274 | *mt-ND5* | a>T | L>L |
| 13282 | *mt-ND5* | a>T | N>I |
| 13300 | *mt-ND5* | c>T | S>L |
| 13333 | *mt-ND5* | c>T | S>F |
| 13340 | *mt-ND5* | t>A | I>M |
| 13350 | *mt-ND5* | a>G | T>A |
| 13355 | *mt-ND5* | c>T | P>P |
| 13371 | *mt-ND5* | c>T | L>L |
| 13391 | *mt-ND5* | a>G | L>L |
| 13407 | *mt-ND5* | a>G | I>V |
| 13409 | *mt-ND5* | c>T | I>I |
| 13418 | *mt-ND5* | a>T | E>D |
| 13445 | *mt-ND5* | t>G | T>T |
| 13449 | *mt-ND5* | c>T | H>Y |
| 13454 | *mt-ND5* | a>T | T>T |
| 13455 | *mt-ND5* | a>G | N>D |
| 13456 | *mt-ND5* | a>G | N>S |
| 13457 | *mt-ND5* | c>T | N>N |
| 13468 | *mt-ND5* | t>C | L>S |
| 13470 | *mt-ND5* | a>G | T>A |
| 13497 | *mt-ND5* | t>C | L>P |
| 13498 | *mt-ND5* | t>G | L>R |
| 13543 | *mt-ND5* | t>C | I>T |
| 13594 | *mt-ND6* | c>T | L>L |
| 13696 | *mt-ND6* | c>T | M>M |
| 13718 | *mt-ND6* | t>A | D>V |
| 13722 | *mt-ND6* | g>A | L>F |
| 13732 | *mt-ND6* | t>C | G>G |
| 13736 | *mt-ND6* | a>G | V>A |
| 13737 | *mt-ND6* | c>T | V>I |
| 13757 | *mt-ND6* | a>T | V>D |
| 13783 | *mt-ND6* | c>T | V>V |
| 13798 | *mt-ND6* | a>G | F>F |
| 13821 | *mt-ND6* | a>G | S>P |
| 13859 | *mt-ND6* | g>A | T>I |
| 13870 | *mt-ND6* | a>T | F>L |
| 13881 | *mt-ND6* | a>G | L>L |
| 13915 | *mt-ND6* | a>G | G>G |
| 13924 | *mt-ND6* | c>T | S>S |
| 13938 | *mt-ND6* | c>A | G>W |
| 13960 | *mt-ND6* | a>T | F>F |
| 13961 | *mt-ND6* | a>G | F>S |
| 13963 | *mt-ND6* | c>T | G>G |
| 13966 | *mt-ND6* | a>G | S>S |
| 13976 | *mt-ND6* | a>G | L>S |
| 13983 | *mt-ND6* | a>G | L>L |
| 13984 | *mt-ND6* | a>G | G>G |
| 14002 | *mt-ND6* | a>G | P>P |
| 14024 | *mt-ND6* | c>T | C>Y |
| 14072 | *mt-TE* | a>T |  |
| 14076 | *mt-TE* | c>T |  |
| 14094 | *mt-TE* | c>T |  |
| 14103 | *mt-TE* | a>G |  |
| 14104 | *mt-TE* | c>T |  |
| 14117 | *mt-TE* | c>T |  |
| 14148 | *mt-CYB* | a>G | T>A |
| 14235 | *mt-CYB* | t>C | W>R |
| 14246 | *mt-CYB* | g>A | G>G |
| 14249 | *mt-CYB* | c>T | S>S |
| 14275 | *mt-CYB* | a>T | Q>L |
| 14279 | *mt-CYB* | c>T | I>I |
| 14283 | *mt-CYB* | a>T | T>S |
| 14297 | *mt-CYB* | a>G | L>L |
| 14302 | *mt-CYB* | t>C | M>T |
| 14312 | *mt-CYB* | a>G | T>T |
| 14320 | *mt-CYB* | c>T | T>T |
| 14335 | *mt-CYB* | c>T | S>L |
| 14344 | *mt-CYB* | c>T | T>M |
| 14345 | *mt-CYB* | a>T | T>T |
| 14366 | *mt-CYB* | t>C | N>N |
| 14377 | *mt-CYB* | t>A | L>P |
| 14395 | *mt-CYB* | c>T | A>V |
| 14407 | *mt-CYB* | c>T | S>L |
| 14438 | *mt-CYB* | c>T | V>V |
| 14447 | *mt-CYB* | c>G | G>G |
| 14476 | *mt-CYB* | a>G | E>G |
| 14495 | *mt-CYB* | a>G | V>V |
| 14499 | *mt-CYB* | c>T | L>L |
| 14505 | *mt-CYB* | t>A | F>I |
| 14506 | *mt-CYB* | t>C | F>S |
| 14508 | *mt-CYB* | g>A | A>T |
| 14516 | *mt-CYB* | a>G | M>M |
| 14527 | *mt-CYB* | t>C | F>S |
| 14537 | *mt-CYB* | c>T | Y>Y |
| 14545 | *mt-CYB* | c>T | P>L |
| 14564 | *mt-CYB* | c>T | F>F |
| 14572 | *mt-CYB* | c>T | A>V |
| 14591 | *mt-CYB* | c>T | L>F |
| 14610 | *mt-CYB* | a>G | I>V |
| 14612 | *mt-CYB* | t>G | I>M |
| 14627 | *mt-CYB* | c>T | V>V |
| 14631 | *mt-CYB* | t>G | W>G |
| 14633 | *mt-CYB* | a>G | W>W |
| 14637 | *mt-CYB* | t>G | W>G |
| 14639 | *mt-CYB* | a>G | W>W |
| 14645 | *mt-CYB* | c>G | G>G |
| 14648 | *mt-CYB* | c>T | F>F |
| 14655 | *mt-CYB* | g>A | D>N |
| 14657 | *mt-CYB* | c>T | D>D |
| 14661 | *mt-CYB* | g>A | A>T |
| 14664 | *mt-CYB* | a>C | T>P |
| 14667 | *mt-CYB* | t>C | L>L |
| 14670 | *mt-CYB* | a>G | T>A |
| 14678 | *mt-CYB* | c>T | F>F |
| 14687 | *mt-CYB* | c>T | F>F |
| 14708 | *mt-CYB* | t>C | I>I |
| 14713 | *mt-CYB* | c>T | A>V |
| 14717 | *mt-CYB* | c>T | A>A |
| 14726 | *mt-CYB* | c>T | I>I |
| 14732 | *mt-CYB* | c>T | H>H |
| 14747 | *mt-CYB* | c>T | H>H |
| 14759 | *mt-CYB* | a>G | S>S |
| 14762 | *mt-CYB* | c>T | N>N |
| 14763 | *mt-CYB* | a>T | N>Y |
| 14765 | *mt-CYB* | c>T | N>N |
| 14782 | *mt-CYB* | c>T | S>L |
| 14826 | *mt-CYB* | g>A | D>N |
| 14837 | *mt-CYB* | t>C | G>G |
| 14842 | *mt-CYB* | t>C | L>P |
| 14844 | *mt-CYB* | a>T | I>F |
| 14861 | *mt-CYB* | c>T | L>L |
| 14863 | *mt-CYB* | t>C | M>T |
| 14864 | *mt-CYB* | a>G | M>M |
| 14888 | *mt-CYB* | c>T | D>D |
| 14891 | *mt-CYB* | a>T | M>I |
| 14892 | *mt-CYB* | c>T | L>L |
| 14897 | *mt-CYB* | a>T | G>G |
| 14905 | *mt-CYB* | a>G | D>G |
| 14915 | *mt-CYB* | a>T | M>I |
| 14924 | *mt-CYB* | t>C | P>P |
| 14948 | *mt-CYB* | t>A | I>M |
| 14960 | *mt-CYB* | a>G | W>W |
| 14971 | *mt-CYB* | t>C | F>S |
| 14973 | *mt-CYB* | g>A | A>T |
| 14978 | *mt-CYB* | c>T | Y>Y |
| 15015 | *mt-CYB* | g>A | V>I |
| 15020 | *mt-CYB* | a>G | L>L |
| 15057 | *mt-CYB* | c>T | P>S |
| 15067 | *mt-CYB* | a>T | H>L |
| 15069 | *mt-CYB* | a>T | T>S |
| 15079 | *mt-CYB* | a>T | Q>L |
| 15092 | *mt-CYB* | a>G | M>M |
| 15130 | *mt-CYB* | t>C | V>A |
| 15134 | *mt-CYB* | c>T | A>A |
| 15143 | *mt-CYB* | t>A | L>L |
| 15151 | *mt-CYB* | c>T | T>I |
| 15191 | *mt-CYB* | c>T | I>I |
| 15214 | *mt-CYB* | c>T | S>L |
| 15239 | *mt-CYB* | t>C | L>L |
| 15285 | *mt-CYB* | c>T | P>S |
| 15303 | *mt-TT* | a>G |  |
| 15308 | *mt-TT* | t>C |  |
| 15375 | *mt-TP* | c>T |  |
| 15381 | *mt-TP* | c>T |  |
| 15388 | *mt-TP* | a>G |  |
| 15400 | *mt-TP* | t>A |  |
| 15404 | *mt-TP* | c>A |  |
| 15414 | *mt-TP* | t>C |  |
| 15440 | Control Region | a>G |  |
| 15536 | Control Region | c>T |  |
| 15573 | Control Region | c>T |  |
| 15617 | Control Region | c>T |  |
| 15702 | Control Region | t>G |  |
| 15787 | Control Region | a>G |  |
| 15884 | Control Region | a>G |  |
| 15962 | Control Region | a>G |  |
| 15967 | Control Region | a>G |  |
| 15969 | Control Region | a>C |  |
| 15972 | Control Region | a>C |  |
| 16099 | Control Region | a>T |  |
| 16165 | Control Region | c>T |  |
| 16213 | Control Region | t>C |  |
| 16218 | Control Region | t>C |  |
| 16248 | Control Region | t>C |  |
| 16270 | Control Region | c>T |  |
| 16272 | Control Region | t>C |  |
| 16276 | Control Region | c>T |  |
| 16294 | Control Region | t>A |  |
