# Supplementary figures and images for: Strong Purifying Selection in Transmission of Mammalian Mitochondrial DNA
Source: PLoS Biol. 2008 Jan 29;6(1):e10. doi: 10.1371/journal.pbio.0060010 (PMC2214808; doi:10.1371/journal.pbio.0060010)

## N2 animals

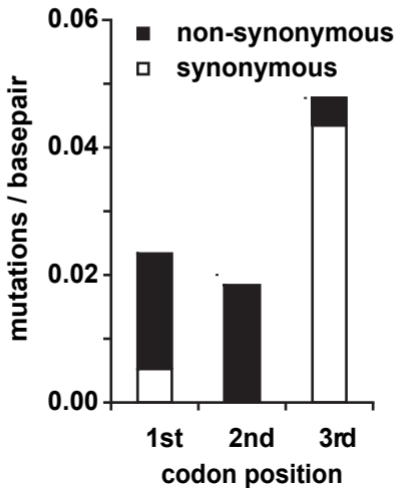

Supplement: Figure S1 — The mutations per base pair observed in N2 animals are plotted by codon position and divided into synonymous (open bars) and nonsynonymous (filled bars) substitutions. A total of 343 mutations were observed: 90 in the first codon position (19 synonymous and 71 nonsynonymous), 70 in the second, and 183 in the third (165 synonymous and 18 nonsynonymous). (1.1 MB AI). [file pbio.0060010.sg001.pdf]
