# Supplementary material for: Strong Purifying Selection in Transmission of Mammalian Mitochondrial DNA
Source: PLoS Biol. 2008 Jan 29;6(1):e10. doi: 10.1371/journal.pbio.0060010 (PMC2214808; doi:10.1371/journal.pbio.0060010)
Supplement: Table S1 — Values calculated for the McDonald-Kreitman (MK) test of neutrality [39] and the accompanying Neutrality Index (NI) [40], comparing the mtDNA mutator line dataset to the mtDNA sequences of M. m. molossinus and M. m. domesticus NZB mouse strains. The mtDNA mutator lines show statistically significant excess of mtDNA nonsynonymous mutations when compared to either outgroup (p-values of a 2 × 2 contingency table using the Fisher exact test are reported). Values are also provided when comparing M. m. molossinus mtDNA to the NZB and C57Bl/6 mtDNA sequences (also statistically significant excess in nonsynonymous substitutions). (30 KB DOC) [file pbio.0060010.st001.doc]

| **Fixed** | **Polymorphic** | **MK ratio** | ***p*** | **NI** |
| --- | --- | --- | --- | --- |
| *M. m. molossinus* | mtDNA mutator strains | 0.1380 | <0.0001 | 7.2459 |
| NZB strain | mtDNA mutator strains | 0.2880 | <0.0001 | 3.4718 |
| *M. m. molossinus* | NZB and C57Bl/6 strains | 0.4791 | 0.0335 | 2.0871 |
