# Supplementary material for: Strong Purifying Selection in Transmission of Mammalian Mitochondrial DNA
Source: PLoS Biol. 2008 Jan 29;6(1):e10. doi: 10.1371/journal.pbio.0060010 (PMC2214808; doi:10.1371/journal.pbio.0060010)
Supplement: Table S2 — Data accompanying Figures 2A and 4A. These values were divided by the number of nucleotides represented by (A) protein-coding genes divided into synonymous and nonsynonymous mutations and (B) tRNAs, rRNAs, and the control region. (32 KB DOC) [file pbio.0060010.st002.doc]

A

|  | **1^st^ codon**  **position** | **2^nd^ codon position** | **3^rd^ codon position** |
| --- | --- | --- | --- |
| **Synonymous** | 35 | - | 330 |
| **Non-synonymous** | 150 | 162 | 40 |
| **Number of positions** | 3803 | 3800 | 3800 |

**B**

|  | **tRNAs** | **rRNAs** | **Non-coding** | **Control region** |
| --- | --- | --- | --- | --- |
| **Number of mutations** | 138 | 192 | 2 | 20 |
| **Number of Positions** | 1535 | 2737 | 25 | 877 |
