# Supplementary material for: Strong Purifying Selection in Transmission of Mammalian Mitochondrial DNA
Source: PLoS Biol. 2008 Jan 29;6(1):e10. doi: 10.1371/journal.pbio.0060010 (PMC2214808; doi:10.1371/journal.pbio.0060010)
Supplement: Table S3 — Data accompanying Figure 3. The expected values were calculated as a proportion of the total observed hits, equally distributed across the number of sites per gene for the two data classes for (A) mtDNA mutator mouse lines and (B) the human mtDB dataset. (73 KB DOC) [file pbio.0060010.st003.doc]

**A Mutator Lines**

|  | ***mt-***  ***ND1*** | ***mt-ND2*** | ***mt-ND3*** | ***mt-ND4L*** | ***mt-ND4*** | ***mt-ND5*** | ***mt-ND6*** | ***mt-CYB*** | ***mt-CO1*** | ***mt-CO2*** | ***mt-CO3*** | ***mt-ATP6*** | ***mt-ATP8*** |
| --- | --- | --- | --- | --- | --- | --- | --- | --- | --- | --- | --- | --- | --- |
| **4-fold degenerate sites** | |  |  |  |  |  |  |  |  |  |  |  |  |
| **number of positions** | 162 | 173 | 55 | 57 | 218 | 281 | 72 | 179 | 275 | 107 | 136 | 118 | 32 |
| **observed** | 17 | 10 | 6 | 7 | 24 | 29 | 8 | 19 | 24 | 13 | 13 | 15 | 5 |
| **expected** | 17 | 18 | 6 | 6 | 22 | 29 | 7 | 18 | 28 | 11 | 14 | 12 | 3 |
|  |  |  |  |  |  |  |  |  |  |  |  |  |  |
| **Other protein coding sites** | | |  |  |  |  |  |  |  |  |  |  |  |
| **number of positions** | 795 | 865 | 293 | 240 | 1160 | 1543 | 447 | 965 | 1270 | 577 | 648 | 563 | 172 |
| **observed** | 50 | 58 | 12 | 12 | 68 | 76 | 18 | 76 | 51 | 22 | 37 | 34 | 13 |
| **expected** | 44 | 48 | 46 | 13 | 64 | 85 | 25 | 53 | 70 | 32 | 36 | 31 | 10 |

**B Human Dataset**

|  | ***mt-***  ***ND1*** | ***mt-ND2*** | ***mt-ND3*** | ***mt-ND4L*** | ***mt-ND4*** | ***mt-ND5*** | ***mt-ND6*** | ***mt-CYB*** | ***mt-CO1*** | ***mt-CO2*** | ***mt-CO3*** | ***mt-ATP6*** | ***mt-ATP8*** |
| --- | --- | --- | --- | --- | --- | --- | --- | --- | --- | --- | --- | --- | --- |
| **4-fold degenerate sites** | |  |  |  |  |  |  |  |  |  |  |  |  |
| **number of positions** | 188 | 189 | 53 | 57 | 256 | 321 | 87 | 119 | 276 | 117 | 137 | 108 | 32 |
| **observed** | 99 | 93 | 21 | 21 | 123 | 161 | 61 | 97 | 130 | 69 | 66 | 55 | 15 |
| **expected** | 98 | 99 | 28 | 30 | 133 | 167 | 45 | 62 | 144 | 61 | 71 | 56 | 17 |
|  |  |  |  |  |  |  |  |  |  |  |  |  |  |
| **Other protein coding sites** | | |  |  |  |  |  |  |  |  |  |  |  |
| **number of positions** | 768 | 853 | 293 | 243 | 1122 | 1494 | 438 | 1022 | 1269 | 561 | 647 | 573 | 178 |
| **observed** | 143 | 165 | 51 | 30 | 169 | 252 | 81 | 215 | 177 | 101 | 122 | 181 | 65 |
| **expected** | 140 | 156 | 53 | 44 | 205 | 272 | 80 | 186 | 231 | 102 | 118 | 105 | 33 |
