# Supplementary material for: Strong Purifying Selection in Transmission of Mammalian Mitochondrial DNA
Source: PLoS Biol. 2008 Jan 29;6(1):e10. doi: 10.1371/journal.pbio.0060010 (PMC2214808; doi:10.1371/journal.pbio.0060010)
Supplement: Table S4 — (89 KB DOC) [file pbio.0060010.st004.doc]

| **Primer**  **Name** | **3' Nucleotide Position** | **Primer Sequence** |
| --- | --- | --- |
| mtF1 | 575 | tgtaaaacgacggccagtagagaactactagccatagc |
| mtR1 | 1198 | caggaaacagctatgacctgcggtactagttctatagc |
| mtF2 | 1101 | tgtaaaacgacggccagtatgaacactctgaactaatcc |
| mtR2 | 1725 | caggaaacagctatgacctactcatactaacagtgttgc |
| mtF3 | 1648 | tgtaaaacgacggccagtagaaagcgttcaagctcaac |
| mtR3 | 2294 | caggaaacagctatgaccactttgacttgtaagtctagg |
| mtF4 | 2135 | tgtaaaacgacggccagtttgacctttcagtgaagagg |
| mtR4 | 2738 | caggaaacagctatgaccagaacactattagggagagg |
| mtF5 | 2685 | tgtaaaacgacggccagtagagaaggttattagggtgg |
| mtR5 | 3311 | caggaaacagctatgaccttgtttctgctagggttgag |
| mtF6 | 3151 | tgtaaaacgacggccagttcactattcggagctttacg |
| mtR6 | 3821 | caggaaacagctatgaccggataaggtgtttaggtagc |
| mtF7 | 3776 | tgtaaaacgacggccagtcaagccctcttatttctagg |
| mtR7 | 4353 | caggaaacagctatgaccgatagtagagttgagtagcg |
| mtF8 | 4260 | tgtaaaacgacggccagtccattccacttctgattacc |
| mtR8 | 4794 | caggaaacagctatgacctgcttatgatagctagggtg |
| mtF9 | 4772 | tgtaaaacgacggccagtcactcatagcaataatagctc |
| mtR9 | 5498 | caggaaacagctatgaccaaaagcatgggcagttacg |
| mtF10 | 5425 | tgtaaaacgacggccagtggaatagtgggtactgcac |
| mtR10 | 6231 | caggaaacagctatgaccgctgatgtaaagtaagctcg |
| mtF11 | 5924 | tgtaaaacgacggccagtctgctcctattatcactacc |
| mtR11 | 6539 | caggaaacagctatgaccttgctcatgtgtcatctagg |
| mtF12 | 6452 | tgtaaaacgacggccagtgatacatactatgtagtagcc |
| mtR12 | 7116 | caggaaacagctatgaccgatatagaggactaaggagc |
| mtF13 | 7045 | tgtaaaacgacggccagttccaacttggtctacaagac |
| mtR13 | 7769 | caggaaacagctatgaccttgatgtatctagttgtgg |
| mtF14 | 7658 | tgtaaaacgacggccagttgtcctagaaatggttccac |
| mtR14 | 8272 | caggaaacagctatgacctttagtttgtgtcggaagcc |
| mtF15 | 8214 | tgtaaaacgacggccagtcatacatttacacctactacc |
| mtR15 | 8814 | caggaaacagctatgacctgtacaataggagtgtggtgg |
| mtF16 | 8745 | tgtaaaacgacggccagtctactcaccaatatcctcac |
| mtR16 | 9496 | caggaaacagctatgaccatgaagataacagtgtacagg |
| mtF17 | 9396 | tgtaaaacgacggccagtttgatgaggatcttactccc |
| mtR17 | 10143 | caggaaacagctatgaccgtaggttgagattttggacg |
| mtF18 | 10079 | tgtaaaacgacggccagtaccatcttagttttcgcagc |
| mtR18 | 10720 | tgtaaaacgacggccagtaatcggttctattccactgc |
| mtF19 | 10647 | tgtaaaacgacggccagtaatcggttctattccactgc |
| mtR19 | 11250 | caggaaacagctatgaccgctagattagctagacttgc |
| mtF20 | 11150 | tgtaaaacgacggccagtcatcatcactcctattctgc |
| mtR20 | 11848 | caggaaacagctatgaccagaatttgattgatgtggtgg |
| mtF21 | 11793 | tgtaaaacgacggccagttcttcattcttctactatccc |
| mtR21 | 12474 | caggaaacagctatgaccgtacttgagtgtagtagtgct |
| mtF22 | 12424 | tgtaaaacgacggccagtaggaaaatcagcacaatttgg |
| mtR22 | 13123 | caggaaacagctatgaccgacaaatcctgcaaagatgc |
| mtF23 | 13015 | tgtaaaacgacggccagtacagctatgtacagcatacg |
| mtR23 | 13665 | caggaaacagctatgaccatgatgttggagttatgttgg |
| mtF24 | 13629 | tgtaaaacgacggccagttactaccatcattcaagtagc |
| mtR24 | 14298 | caggaaacagctatgacctctgatgtgtagtgtatggc |
| mtF25 | 14209 | tgtaaaacgacggccagtcactcattcattgacctacc |
| mtR25 | 14799 | caggaaacagctatgaccgtatagtaggggtgaaatgg |
| mtF26 | 14701 | tgtaaaacgacggccagtgctttccacttcatcttacc |
| mtR26 | 15314 | caggaaacagctatgacctcatttcaggtttacaagacc |
| mtF27 | 15161 | tgtaaaacgacggccagtcttatcttaacctgaattggg |
| mtR27 | 15879 | caggaaacagctatgacccacagttatgttggtcatgg |
| mtF28 | 15794 | tgtaaaacgacggccagtgaaactttatcagacatctgg |
| mtR28 | 113 | caggaaacagctatgacctctatggaggtttgcatgtg |
| mtF29 | 74 | tgtaaaacgacggccagttgtatcccataaacacaaagg |
| mtR29 | 657 | caggaaacagctatgaccgctgaattagcaagagatgg |
|  |  |  |
| **Additional Sequencing Primers** | |  |
| mtF9b | 5200 | aatggcggtagaagtcttag |
| mtR9b | 5175 | tttttcggcggtagaagtag |
| mtF17b | 9844 | aaaaaaattaatgatttcgactc |
| mtR17b | 9798 | ttttaaactaattaccatttactctg |
